# Polymer Coated Iron–Boron and Gold–Iron Alloy Nanoparticles for Magnetic Resonance Imaging and Near Infrared Photothermal Applications

**DOI:** 10.1021/acsanm.5c04740

**Published:** 2025-12-11

**Authors:** Michael Bissoli, Alessandro Negri, Asya Zerbato, Denis Badocco, Paolo Pastore, Marta Filibian, Francesca Brero, Silvia Megalizzi, Alessandro Lascialfari, Nicola Greco, Pasquina Marzola, Vincenzo Amendola

**Affiliations:** 1 Department of Chemical Sciences, 9308 University of Padova, Padova 35131, Italy; 2 Department of Engineering for Innovation Medicine, 19051 University of Verona, Verona 37134, Italy; 3 Centro Grandi Strumenti, 19001 University of Pavia, Pavia 27100, Italy; 4 National Institute for Nuclear Physics, Pavia Unit, Pavia 27100, Italy; 5 Department of Physics, University of Pavia, Pavia 27100, Italy

**Keywords:** laser ablation, nanoalloy, iron nanoparticles, photothermal heating, MRI

## Abstract

Iron alloy nanoparticles (IA NPs) are appealing tools for nanomedicine because of their composition-dependent properties, including magnetism, near-infrared (NIR) absorption, radiosensitization, and biodegradability. However, NP stability in biological environments is a limiting factor in their use. The effective surface modification of IA NPs using hydrophilic biocompatible molecules is the only strategy to optimize the half-life in blood circulation, biodistribution, cellular absorption, and bioaccumulation. Here, IA NPs based on iron–boron (Fe–B) and gold–iron (Au–Fe) were surface stabilized using various polyethylene glycol derivatives or polyvinylpyrrolidone, which were then combined with silanes and citric acid to determine the best formulation in terms of nanometric size, biocompatible surface chemistry, and stability in biological fluids. The IA NPs were synthesized in a single step utilizing laser ablation in liquid, enabling the exploration of different types of surface coatings selected specifically for their affinity with the elements comprising the nanoalloys. Then, the most stable Fe–B and Au–Fe formulations were tested as contrast agents for magnetic resonance imaging (MRI) and as sensitizers for photothermal heating using NIR light, a property not previously investigated in these IA NPs but of interest for their application in photothermal therapy (PTT). Notably, the most stable Fe–B NPs performed well as MRI contrast agents and absorbed substantially in the NIR spectrum, making them viable candidates for use as photosensitizers. Preliminary *in vitro* tests confirmed the positive prospects of polymer-stabilized Fe–B NPs for PTT.

## Graphical abstract

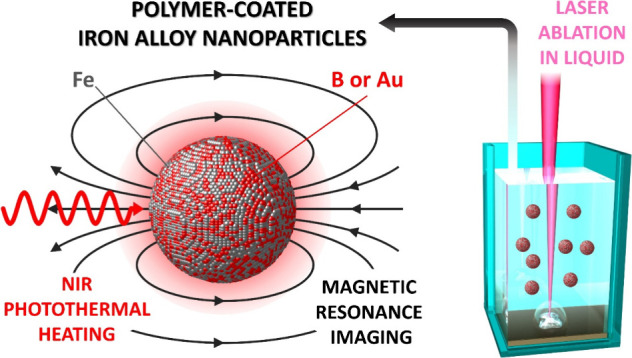

## 1 Introduction

Iron alloy nanoparticles (IA NPs) have gained widespread attention in recent decades because of their potential applications in biomedicine.^1,2^ Iron (Fe) is valued for its desirable properties, including magnetism. Furthermore, unlike other magnetic elements such as cobalt and nickel, Fe is biocompatible.^2,3^ This allows the exploitation of IA NPs as contrast agents for magnetic resonance imaging (MRI).^2,4,5^ MRI is one of the most effective noninvasive diagnostic techniques clinically available because it delivers exceptional spatial and temporal resolution, showcasing remarkable inherent contrast in soft tissues without the use of ionizing radiation or radiotracers.^5,6^ Typically, Fe-based NPs produce negative contrast (they appear dark) in MRI images by shortening the proton spin transverse relaxation time (*T*_2_), thus acting as *T*_2_-weighted contrast agents.^3,5–7^ Additionally, IA NPs with high magnetization are exploitable for magnetic hyperthermia therapy (MHT).^8^ MHT has a lower adverse impact than chemotherapy or radiotherapy, achieving localized temperature elevation in the vicinity of a tumor by applying an alternating magnetic field with appropriate amplitude and frequency.^8^ Using magnetic NPs that selectively accumulate within tumor tissues, the effective applicability of MHT is substantially improved in terms of safety, invasiveness, tissue-specificity, and overall efficacy.^8^ Nevertheless, achieving the required MHT performance for Fe-based NPs remains a challenge because they tend to oxidize or require chemical stabilization in their metallic form by alloying with other nonmagnetic elements.^8,9^

An alternative approach for cancer thermal therapy that has received great consideration in the context of nanomedicine is photothermal therapy (PTT).^10^ PTT is based on a light-to-heat energy conversion mechanism, exploiting NPs as photosensitizers.^11^ Additionally, optical radiation can be precisely controlled in terms of time and spatial resolution, ensuring accurate and controlled treatment administration.^10–12^ However, one main limitation of PTT is related to light absorption and diffusion by biological tissues; thus, near-infrared (NIR) radiation is adopted because it can penetrate the skin and other tissues with minimal absorption by water or hemoglobin.^10–12^ PTT using IA NPs has been much less investigated than MHT because traditional PTT nanomaterials are noble metals, semiconductors, or bidimensional layered compounds with nonmagnetic properties.^10,11,13^ In fact, the photosensitization properties of IA NPs are mostly unknown. Additionally, recent studies of iron–boron (Fe–B)^14^ and gold–iron (Au–Fe)^15^ NPs have demonstrated that when Fe is alloyed with other elements to add specific functions, the resulting IA NPs can exhibit innovative or enhanced properties and tailored functionalities that are not found in single-element nanomaterials. Fe–B NPs can also be used for boron neutron capture therapy guided by MRI.^14,16,17^ Au–Fe NPs can be exploited for computed X-ray tomography because of the high X-ray attenuation cross section of Au,^15^ as well as for MRI. Furthermore, both IA NPs behave as biodegradable nanomedicines in a physiological environment.^14,15^ This property, which is composition dependent in Au–Fe, is crucial for nanomedicine applications because of the substantial challenge of prolonged biopersistence arising from limited or null degradability and the resultant accumulation within the human body.^15,18–20^

Nonetheless, various physicochemical attributes play a role in the development of IA NPs, with particular regard to biocompatibility, water dispersibility, and colloidal stability.^21–23^ The method used to synthesize IA NPs is important because it determines unique properties such as size, shape, composition, surface chemistry, and stability in aqueous media. In particular, the surface chemistry of IA NPs can considerably enhance their interactions with biological systems, thereby influencing and advancing their performance. Achieving long-term stability for Fe–B and Au–Fe NPs in biological fluids can be challenging because of several factors. Depending on the ionic strength, pH, and type of biological components present in the medium, Fe–B and Au–Fe NPs tend to agglomerate or form clusters in aqueous media because of attractive forces between particles, causing instability and eventually aggregation.^21–23^ To increase the stability and dispersibility of these NPs in aqueous media, it is essential to modify the IA NP surfaces with hydrophilic molecules (biocompatible polymers) and/or other stabilizers (e.g., citric acid (CA)) to create a surface coating and change the surface charge.^1,2,14,15,24,25^ Surface modification using biocompatible polymers that expose specific functional groups at the end of the polymeric chain can improve NP biocompatibility by reducing their toxicity, enabling NP delivery to specific cells or tissues, and dramatically influencing corrosion resistance in body fluids.^1,2,14,15,24^ These biocompatible polymers include polyethylene glycol (PEG) with various functionalizations, such as thiol,^15^ amino,^26^ or carboxylic^27^ groups, in a single or multiarmed configuration, as well as polyvinylpyrrolidone (PVP),^14^ poly­(vinyl alcohol) (PVA),^28^ or silane matrices.^29,30^ Other types of stabilizing agents described in the literature include phosphonates^31^ and citrate groups,^32,33^ which have a chelating effect on Fe atoms and can prevent aggregation while maintaining NP dispersion in solution by creating a protective layer around the NPs.

Herein, we used a one-step synthetic approach based on laser ablation in liquid (LAL) to realize two types of IA NPs: Fe–B and Au–Fe enveloped in a shell of biocompatible polymers. These are candidates as nanomedicine agents because they combine the magnetic properties of Fe with the advantages of Au or B.^14,15^ LAL enables the production of bimetallic nanoalloys coated with organic molecules during the synthesis (*in situ*) process.^34–36^ This synthetic approach has the advantage of being simple to implement, cost-effective, compatible with green chemistry principles, and does not require the introduction of unwanted chemical contaminants, starting only with bulk metal components and a solution of the biocompatible polymer in a pure solvent.^34,35,37,38^ The stability of the Fe–B and Au–Fe NPs was evaluated as a function of surface coating to identify the optimal formulation. Then, two relevant properties of the IA NPs for nanomedical applications—MRI contrast agent efficacy and photothermal efficacy—were investigated and compared. In this approach, Fe–B and Au–Fe alloy NPs were evaluated for the first time for use in PTT and in combination with MRI, demonstrating their excellent prospects for further development in this direction.

## 2 Materials and Methods

### 2.1 Chemicals

Fe­(65 at%)–B­(35 at%) and Au­(25 at%)–Fe­(75 at%) metal targets were purchased from MaTeck GmbH. PVP (40 kDa), CA, tribasic sodium citrate, and ethanol (HPLC grade) were purchased from Sigma-Aldrich. PEG–thiol (PEG–SH, 5 kDa), amine–PEG–thiol (NH_2_–PEG–SH, 5 kDa), and 4 Arm PEG–COOH (4 Arm PEG–ASA, 20 kDa) were purchased from Laysan Bio. SH–PEG–COOH (HS–C_2_H_4_–CONH–PEG–O–C_3_H_6_–COOH, 3 kDa) was purchased from Rapp Polymere. Acetone (anhydrous) was purchased from VWR. Acetone (HPLC grade) was purchased from Carlo Erba. Reference Au NPs coated with PEG–SH (Au@PEG–SH) were produced via LAL in water with 10^–4^ M sodium chloride, as reported previously.^39^

### 2.2 Synthesis of Fe–B NP Samples

Fe–B NPs were synthesized via LAL according to a modification of a previously established procedure.^14^ LAL was performed using 1064 nm (6 ns, 50 Hz) laser pulses focused to 5.0 J/cm^2^ through a f = 10 cm lens on a bulk Fe–B plate immersed in a cell containing an acetone solution under an argon (Ar) atmosphere. For the Fe–B@PVP + CA sample (Fe–B@PVP–CA), LAL was performed as described above in a solution containing 0.1 mg/mL of PVP and 10^–3^ M CA in anhydrous acetone. For the Fe–B@4 Arm PEG–ASA + CA sample (Fe–B@PEG–ASA–CA), LAL was performed as described above in a solution containing 0.1 mg/mL of 4 Arm PEG–ASA and 10^–3^ M CA in anhydrous acetone. For the Fe–B@PVP + 4 Arm PEG–ASA + CA sample (Fe–B@PVP–PEG–ASA–CA), LAL was performed as described above in a solution containing 0.05 mg/mL of PVP, 0.05 mg/mL of 4 Arm PEG–ASA, and 10^–3^ M CA in anhydrous acetone.

The resulting NP dispersions were all concentrated using a rotavapor at 30 °C and subsequently dried under a nitrogen flow. The powder was resuspended in 5 mL of deionized water and dialyzed (10 kDa, Sartorius Vivaspin) to eliminate residual salts and reaction byproducts. Finally, the NPs were redispersed in 5 mL of deionized water. The general procedure is summarized in Figure 1A.

**Figure 1:**
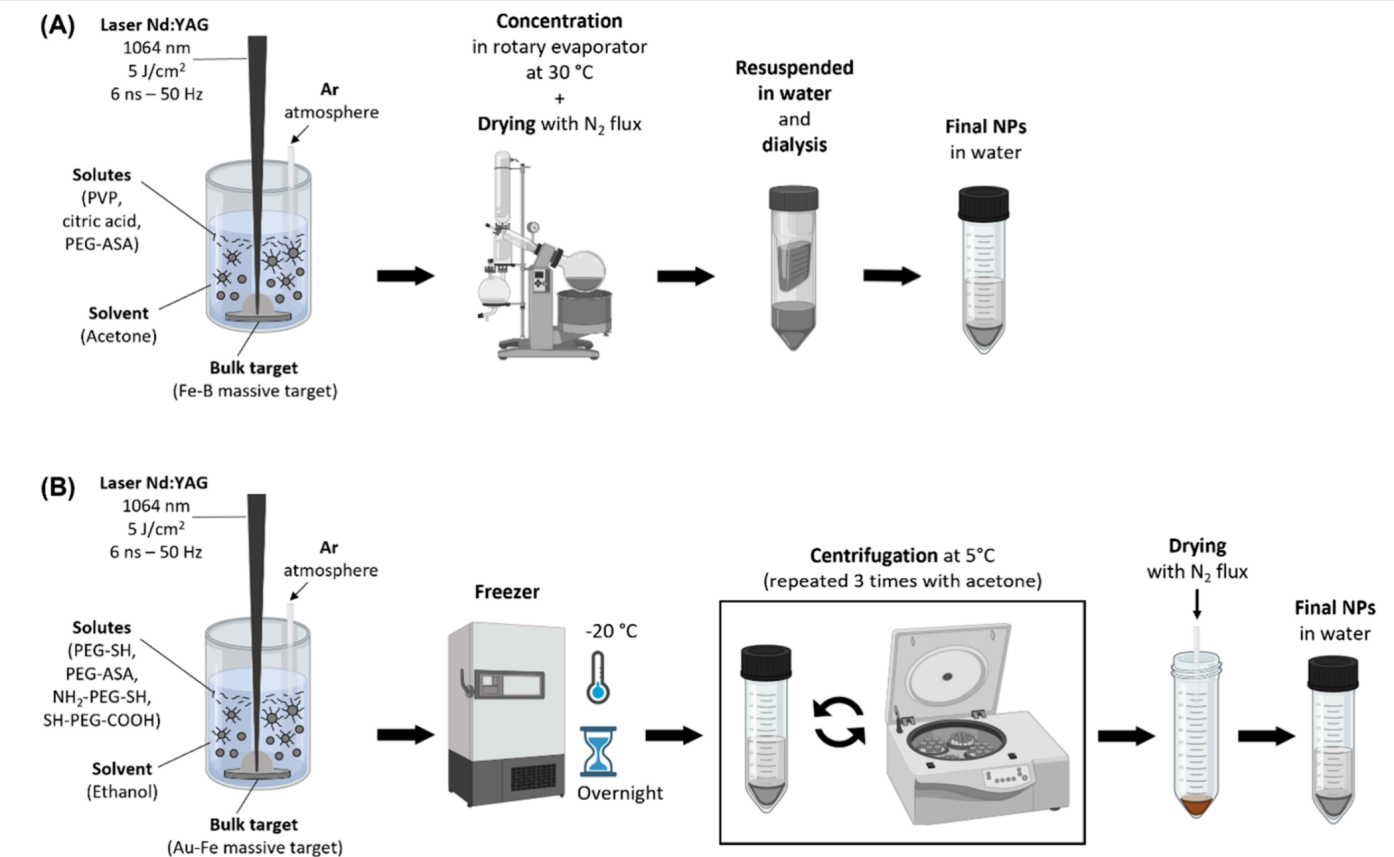
Sketch of the synthetic procedure for polymer coated Fe–B (A) and Au–Fe (B) IA NPs. Created using Biorender.com.

Sedimentation-based separation (SBS)^40^ of the Fe–B@PEG–ASA–CA sample was performed in 1.5 mL tubes via centrifugation at 700 and 1600 rcf for 20 min.

### 2.3 Synthesis of Au–Fe NP Samples

Au–Fe NPs were synthesized via LAL according to a modification of a previously established procedure.^15,39^ LAL was performed using 1064 nm (6 ns, 50 Hz) laser pulses focused to 5.0 J/cm^2^ with a f = 10 cm lens on a bulk Au­(25 at%)–Fe­(75 at%) plate immersed in a cell containing an ethanol solution under an Ar atmosphere. For the Au–Fe@PEG–SH sample, LAL was performed in a solution containing 0.088 mg/mL of PEG–SH. For the Au–Fe@4 Arm PEG–ASA sample (Au–Fe@PEG–ASA), LAL was performed in a solution containing 0.088 mg/mL of 4 Arm PEG–ASA. For the Au–Fe@PEG–SH + 4 Arm PEG–ASA sample (Au–Fe@PEG–SH–ASA), LAL was performed in a solution containing 0.044 mg/mL of PEG–SH and 0.044 mg/mL of 4 Arm PEG–ASA. For the Au–Fe@PEG–SH + NH_2_–PEG–SH (Au–Fe@PEG–SH–NH_2_), LAL was performed in a solution containing 0.044 mg/mL of PEG–SH and 0.044 mg/mL of NH_2_–PEG–SH. For the Au–Fe@PEG–SH + SH–PEG–COOH sample (Au–Fe@PEG–SH–COOH), LAL was performed in a solution containing 0.044 mg/mL of PEG–SH + 0.044 mg/mL of SH–PEG–COOH. For the Au–Fe@NH_2_–PEG–SH + SH–PEG–COOH sample (Au–Fe@PEG–NH_2_–COOH), LAL was performed in a solution containing 0.044 mg/mL of NH_2_–PEG–SH and 0.044 mg/mL of SH–PEG–COOH. The resulting NP dispersions were stored at – 20 °C overnight, recovered via centrifugation (1000 rcf for 1 h at 5 °C), and washed three times with acetone (HPLC grade) via centrifugation (1000 rcf for 1 h at 5 °C) to remove unbound PEG and other synthesis byproducts. Finally, the NPs were dried under a nitrogen flow and resuspended in 5 mL of deionized water. The general procedure is summarized in Figure 1B.

### 2.4 Characterization

Inductively coupled plasma-assisted mass spectrometry (ICP–MS) analysis was performed using an Agilent Technologies 7700x ICP–MS, using the operating conditions and data acquisition parameters previously described.^14,15^ The instrument was equipped with an octupole collision cell operating in kinetic energy discrimination mode, which is used for the removal of polyatomic and Ar-based interferences. Optimal performance was attained using the collision cell in He mode. For sample digestion, 0.5 g of the solution was accurately weighed and digested with 0.8 g of nitric acid (69%) at 100 °C for 2 h. The resulting solution was diluted with the same solvent used for calibrations. Each digested solution was diluted to obtain two concentrations, and each solution was measured three times using the ICP–MS.

Ultraviolet–visible (UV–Vis)–NIR spectra were recorded using a Jasco V770 spectrophotometer with 2 mm optical path quartz cells.

Dynamic light scattering (DLS) was performed using a Malvern Zetasizer Nano ZS instrument equipped with a solid-state laser (633 nm). All measurements were performed at a 173° backscattered angle (NIBS default) in ZEN0112 cells with 15 runs of 10 s each, repeated 3 times. The size was calculated from the size histograms as the average, accompanied by the standard deviation (SD). Z-potential analysis was performed using the same DLS instrument in a DTS1070 cuvette.

The Fourier-transform infrared (FTIR) spectra of the powder samples deposited on a KBr window were collected using a PerkinElmer 1720X spectrometer.

Transmission electron microscopy (TEM) analysis was performed using a FEI Tecnai G2 12 transmission electron microscope operating at 100 kV and equipped with a TVIPS CCD camera. The samples for TEM analysis were prepared by evaporating NP suspensions on a copper grid coated with an amorphous carbon holey film. A minimum of 500 NPs was used to obtain the particle size distribution using ImageJ software. High-resolution TEM (HRTEM) was performed using a scanning transmission electron microscope JEOL JEM F200 operated at 200 kV. Elemental analysis mapping was performed using a JEOL 100 mm^2^ silicon drift energy-dispersive X-ray (EDX) spectrometer.

X-ray powder diffraction (XRD) patterns were collected from powder samples deposited on silicon zero-background substrates with a Panalytical XPert 3 powder diffractometer equipped with a copper tube (40 kV, 40 mA), a BBHD mirror, a spinner, and a PlXcel detector. Crystalline phase identification and Rietveld analysis were executed using Bruker DIFFRAC.EVA software, the TOPAS Academic V6 (Bruker AXS), and the COD database (FeB: COD 4003012; B_2_O: COD 1510794; Fe_2_B: COD 1511152; Fe: COD 7204904; Au–Fe: 0053764).

Photothermal heating experiments were performed on phantoms of the IA NPs (0.091 ± 0.009 mg_Fe/mL for both Au–Fe@PEG–SH–COOH and Fe–B@PVP–PEG–ASA–CA NPs) in agarose (0.5 wt %) using a 785 nm continuous wave laser (CNI MDL-III-785–500 mW, maximum power of 534 mW, laser spot of 8 mm × 5 mm, maximum laser intensity of 1.335 W/cm^2^). A thermal camera model FLIR E5 was used to capture the calibrated digital thermographic infrared images of the heated samples over time. For Fe and B release over time, the Fe–B@PVP–PEG–ASA–CA NPs were dispersed in distilled water at the same concentration as the other photothermal experiments and analyzed after 0, 1800, or 3600 s, either under 534 mW laser irradiation (1.334 W/cm^2^) or in the dark. Each solution was centrifuged at 30,000 rcf for 20 min to completely remove the NPs, and the supernatant was analyzed using ICP–MS.

Magnetic resonance images were acquired using a Bruker Pharmascan system operating at 7 T (Bruker Biospin, Ettlingen, Germany). Nuclear magnetic resonance (NMR) relaxometry measurements were performed using a Tecmag Apollo Fourier-transform NMR system coupled with a Bruker MAGNET B-E 25 electromagnet operating at 1.5 T (Billerica, MA, USA). The samples were dispersed in agarose phantoms (1 wt %) via serial dilution. The transversal relaxivity (r_2_, mM_Fe^–1^s^–1^) was calculated as the slope of the best linear fit of the relaxation rate (1/T_2_) versus Fe concentration. For the NMR relaxometry measurements at 1.5 T, T_1_ and T_2_ values were determined using a standard SR pulse sequence and a CPMG sequence, respectively. At 7 T, the T_2_ maps of the Au–Fe NPs in phantoms were acquired using a Multi Slice Multi Echo (MSME) pulse sequence with the following parameters: TR = 12,000 ms, TE ranging from 6.5 to 292.5 ms; FOV = 55 × 36 mm, and slice thickness = 2 mm. The T_2_ maps of Fe–B NPs in phantoms were acquired using a 2D MSME pulse sequence with the following parameters: TR = 2000 ms, TE ranging from 6.5 to 170.43 ms, number of echoes = 25, FOV = 55 × 55 mm^2^, matrix size = 128 × 128, in-plane resolution = 0.430 × 0.430 mm^2^, slice thickness = 1 mm, and NEX = 4.

### 2.5 *In* *Vitro* Cytotoxicity

The metabolic activity of A375 human melanoma cells (ATCC, Manassas, VA, USA) and WI-26 human lung fibroblasts (ATCC, CCL-75) was evaluated using the MTT assay. For the cytotoxicity assessment of Fe–B NPs, 5000 cells/well were seeded in a 96-well plate and incubated for 24 h at 37 °C and 5% carbon dioxide in Dulbecco’s modified Eagle’s medium (Gibco, Thermo Fisher Scientific, MA, USA) supplemented with 10% fetal bovine serum (FBS), 1% penicillin/streptomycin (P/S), and 1% l-glutamine. After the initial incubation, the medium was replaced with fresh medium containing Fe–B NPs at concentrations ranging from 200 to 12.5 μg_Fe/mL. After 24 and 48 h of incubation, the medium was removed, and 100 μL of MTT solution (5 mg/mL) was added to each well and incubated for an additional 4 h. The resulting formazan crystals were dissolved in 100 μL of dimethyl sulfoxide, and absorbance was measured at 570 nm using an HTX microplate reader (BioTek Instruments, Winooski, VT, USA). Cell viability (CV%) was calculated using the equation CV% = (OD_sample/OD_control) × 100, where OD is the optical density. The results are presented as relative metabolic activity compared to cells without NPs, which are considered 100% vital.

### 2.6 *In Vivo* MRI Experiments

The biodistribution of Fe–B NPs (Fe–B@PVP–PEG–ASA–CA) was evaluated *in vivo* using three normal nude homozygote female mice (8–10 weeks old; Envigo, Bresso, Italy). Mice were anesthetized with 1.5% isoflurane in a mixture of oxygen and air, placed in a heated animal bed, and inserted into a 40 mm internal diameter MRI coil. MRI scans were performed using a Bruker Biospin 7 T scanner (Bruker Biospin, Ettlingen, Germany). Fe–B NPs were administered intravenously via tail vein injection at a dose of 4.0 mg_Fe/kg. Imaging was conducted preinjection and at 1, 5, 24, 48, and 72 h postinjection to monitor biodistribution. A T_2_-weighted rapid acquisition with a relaxation enhancement fat-suppressed sequence was used with the following parameters: TR = 2500 ms, TE_eff = 35 ms, FOV = 35 × 35 mm^2^, matrix size = 200 × 200, slice thickness = 0.5 mm, and NEX = 2. To allow for signal normalization, a reference tube filled with water was placed in the imaging coil alongside the animal. The signal intensity of each organ (SI_organ) was normalized to the signal intensity of the reference tube (SI_standard), and the results are presented as normalized signal intensity (SI_organ/SI_standard). Additionally, T_2_ relaxation times were quantified using a 2D Multi Spin Echo sequence, with TR = 3500 ms and TE values ranging from 8 to 80 ms (echo spacing = 8 ms, 10 echoes). Axial images were acquired with a matrix size of 156 × 156, FOV = 35 × 35 mm^2^, in-plane resolution = 0.224 × 0.224 mm^2^, 30 slices, slice thickness = 1 mm, and NEX = 1. T_2_ decay maps were computed using pixel-wise monoexponential fitting. The biocompatibility of the NPs was confirmed by monitoring body weight and clinical signs (e.g., spontaneous movement, head posture, and social behavior) for 24 h postinjection. The absence of acute toxicity was assessed via observations of skin, fur, eyes, and potential tremors or convulsions. The experimental protocol was approved by the Italian Ministry of Health (protocol 56DC9.82) and the local ethical committee, in accordance with Italian (D.L. March 4, 2014 no. 26) and EU (2010/63/EU) regulations.

### 2.7 Histological Analysis after IV Administration

After *in vivo* administration of Fe–B NPs via intravenous injection, histological analyses were performed on different organs to confirm the biosafety of the NPs. Mice were sacrificed 72 h postadministration, and the heart, liver, spleen, kidneys, and lungs were dissected and fixed in 4% paraformaldehyde overnight at 4 °C. All the samples were dehydrated in solutions containing increasing amounts of ethanol (from 70% to 100%), cleared in xylene, and embedded in paraffin for microtome sectioning. Sections (10 μm thick) were collected from all the organs. The sections were rehydrated and incubated with Perl’s Prussian blue (PB) solution (5% hydrochloric acid and 5% potassium ferrocyanide) for 30 min and counterstained with hematoxylin and eosin (H&E). The sections were then dehydrated using increasing grades of ethanol, cleared in xylene, and coverslipped with Entellan (Merck, Darmstadt, Germany). Images of the sections were acquired using an Olympus APXVIEW APX100 optical microscope (Olympus Italia S.r.l., Segrate, MI, Italy) and examined using a histological atlas.

### 2.8 NP Uptake

A375 cells (1 × 10^5^) were seeded onto 13 mm coverslips, placed in 24-well plates, and left to attach for 24 h at 37 °C. Next, Fe–B NPs were added to the culture medium at a concentration of 50 μg_Fe/mL. Cells were incubated with Fe–B NPs for 24 and 48 h, washed with PBS, and fixed with 4% PFA. PB staining was performed to evaluate the presence of Fe within the cells. Cells were incubated for 30 min with 10% potassium ferrocyanide and 20% hydrochloric acid (mixed 1:1), followed by counterstaining with nuclear fast red for 5 min. Imaging was performed using a light microscope (Olympus BXS1, Evident Corporation, Tokyo, Japan).

### 2.9 Ultrastructural Morphology

TEM was used to confirm internalization of the Fe–B NPs. Cells were pelleted after 24 and 48 h of incubation with NPs and fixed in 2.5% glutaraldehyde for 2 h. All samples were postfixed in 1% osmium tetroxide, dehydrated, and embedded in an Epon–Araldite mixture. Semithin sections were cut and examined, and ultrathin sections were cut at a 65 nm thickness. TEM images were acquired using a Philips Morgagni 268 D (FEI Company, Eindhoven, NL, USA) operating at 80 kV, equipped with a Mega View II Camera (Clympus, Tokyo, Japan).

### 2.10 Photothermal Treatment

To evaluate the safety of laser irradiation, A375 cells not exposed to Fe–B NPs were subjected to 785 nm irradiation at different laser powers (500, 250, 200, 150, 100, and 50 mW, corresponding to 1.25, 0.625, 0.5, 0.375, 0.25, and 0.125 W/cm^2^) for 10 min. After irradiation, the cells were returned to standard culture conditions for 24 h, after which cell vitality was assessed using the MTT assay. To investigate the therapeutic efficacy of Fe–B NPs under photothermal treatment, A375 cells were incubated with Fe–B NPs at a concentration of 50 μg_Fe/mL for 24 and 48 h. The cells were then irradiated using a 785 nm laser at a fixed power of 200 mW (0.5 W/cm^2^) for either 10 or 30 min. After irradiation, the cells were incubated under standard culture conditions for 24 or 48 h, after which cell vitality was assessed using the MTT assay.

## 3 Results and Discussion

### 3.1 Fe–B NPs

The three Fe–B NP samples obtained after the synthesis and purification procedures were initially investigated using UV–Vis–NIR spectroscopy (Figure 2A). The Fe–B NPs samples displayed featureless spectra with optical extinction throughout the 200–1200 nm spectral range, which continuously increased toward shorter wavelengths, with only minor differences observed among the three samples. As expected, the absorption spectrum profiles did not change with the type of coating, indicating that the polymer type did not affect the IA NP composition. In fact, *in situ* investigations of the ultrafast NP formation in LAL have shown that polymers are only involved in the later stage of NP agglomeration and coalescence in the liquid solution.^41^ However, based on previous studies on Fe–B NPs,^14^ the blue region around 450 nm can be considered as an indicator of NP concentration because it is scarcely affected by molecular compound absorption and light scattering from large NP aggregates, which dominates at long wavelengths such as NIR.^42^ Therefore, the absorbance at 450 nm was used as an end point for the synthesis yield, in this case suggesting that the largest yield was achieved for the Fe–B@PEG–ASA–CA sample (bottom of Figure 2B). Additionally, the ratio of absorbance at 450 nm to that at 1200 nm (absorbance@450 nm/absorbance@1200 nm, top of Figure 2B) is higher for NPs that are effectively dispersed in the liquid because of the better efficacy of the surface functionalization. This absorbance ratio was higher in the Fe–B@PVP–CA sample. The Fe–B@PVP–PEG–ASA–CA was intermediate in both end points, offering the best compromise of yield and colloidal dispersion.

**Figure 2:**
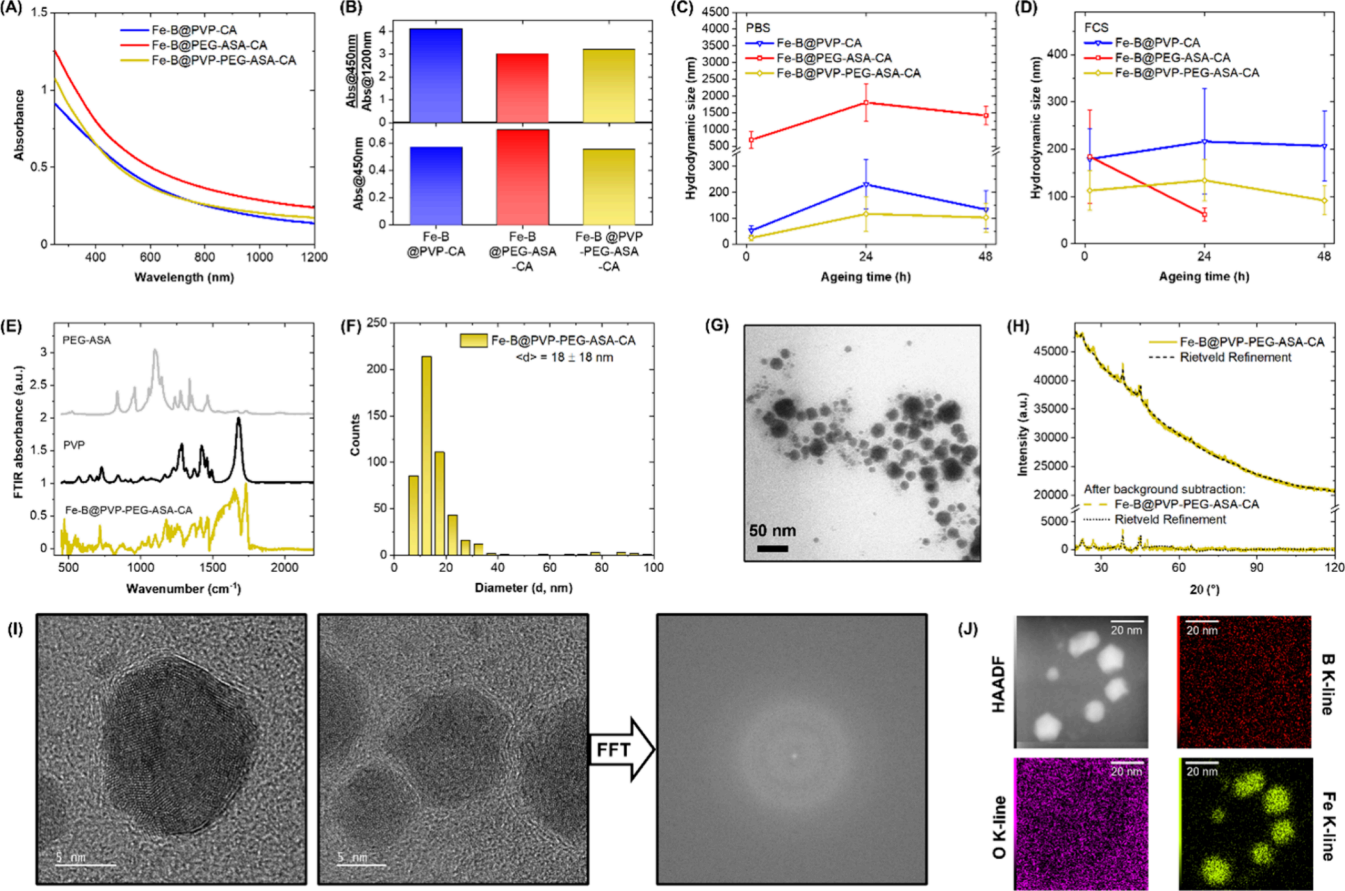
(A) UV–vis–NIR spectra of the three Fe–B NP formulations: PVP–CA (blue), PEG–ASA–CA (red), and PVP–PEG–ASA–CA (yellow). (B) Bottom: absorbance@450 nm for the three samples, indicating their concentration and synthesis yield. Top: absorbance@450 nm/Absorbance@1200 nm ratio, indicating the effective dispersion of the Fe–B NPs versus their aggregation among the three samples. (C, D) Hydrodynamic size of the three Fe–B NP formulations after aging from 1 to 48 h in PBS (C) or FCS (D). (E) FTIR spectra of Fe–B@PVP–PEG–ASA–CA (yellow), a reference PVP (black), and 4 Arm PEG–ASA (gray) powders. (F, G) Fe–B@PVP–PEG–ASA–CA size distribution obtained using TEM analysis of >500 NPs (F) and representative TEM image (G). (H) XRD diffractogram of Fe–B@PVP–PEG–ASA–CA (gold line) and Rietveld refinement (black dashed line). (I) HRTEM and related FFT of Fe–B@PVP–PEG–ASA–CA NPs. (J) EDX mapping indicating homogeneous distribution of Fe in the Fe–B NPs and the absence of an oxygen signal. The B signal is below the detection limit, thus indicating that no B segregation or single B NPs are present.

The stability and hydrodynamic size of the three samples were measured using DLS in phosphate-buffered saline (PBS, Figure 2C) and fetal calf serum (FCS, Figure 2D) solutions, which both simulate the physiological conditions in the body.^14,15,43^ The hydrodynamic size of the three samples was monitored over an aging period from 1 to 48 h at 37 °C, with the Fe–B@PVP–PEG–ASA–CA showing a better response than the other two samples. The hydrodynamic size of the Fe–B@PVP–PEG–ASA–CA sample changed from 24 ± 10 nm to 103 ± 56 nm after 48 h in PBS and from 113 ± 41 nm to 92 ± 31 nm after 48 h in FCS. Under the same conditions, the Fe–B@PVP–CA sample changed from 53 ± 19 nm to 133 ± 73 nm after 48 h in PBS and from 179 ± 64 nm to 207 ± 74 nm after 48 h in FCS. The Fe–B@PEG–ASA–CA sample changed from 689 ± 261 nm to 1414 ± 269 nm after 48 h in PBS and from 184 ± 98 nm to 62 ± 13 nm after 24 h in FCS. In this case, the experiment was interrupted after 24 h because the remarkable size reduction was associated with irreversible sedimentation caused by the ineffective polymer coating on the NPs.

The z-potential for the Fe–B@PVP–PEG–ASA–CA formulation was – 14.1 ± 2.4 mV in PBS. This value was not particularly high compared to the reference value for stable negatively charged NPs, which should be above – 20/–30 mV.^44,45^ Nevertheless, this result indicates an electrostatic contribution to colloid stability, presumably attributable to the carboxylic groups in the NP polymeric coating, which are negatively charged in PBS.^46^

The successful coating of the Fe–B@PVP–PEG–ASA–CA sample was further confirmed using FTIR spectroscopy (Figure 2E), which showed distinctive PVP and PEG–ASA signatures when compared to the powder samples of these polymers. Because the NPs were collected after repeated dialysis cycles with pure water (Figure 1), the unbound polymer was completely removed during this step, indicating that the polymer detected using FTIR was bound to the NPs. The most evident vibrational bands for PVP were C = O stretching at ∼ 1700 cm^–1^, C–H bending at ∼ 1400 cm^–1^, and CH_2_ bending at 700 cm^–1^.^47^ Conversely, PEG exhibited a vibrational fingerprint of C–O–C stretching at 1100 cm^–1^, vibrational progression in the 800–1500 cm^–1^ range, and alkyl C–H stretching bands at high wavenumbers (2900 cm^–1^).^15,48^ However, it is possible that the PEG and CA mixture was partly degraded *in situ* by the laser ablation process, resulting in the formation of graphitic nanostructures catalyzed by the IA NPs.^49,50^ This is consistent with the bands at ∼ 1540 and ∼ 1620 cm^–1^, which correspond to the C = C and C = O groups in partially amorphous or oxidized graphitic materials.^51^

Next, the morphology and size of the NPs in the Fe–B@PVP–PEG–ASA–CA sample were analyzed using TEM imaging (Figure 2F and G). The Fe–B NPs had a spherical shape and an average size of 18 ± 18 nm. Although their morphology was well-defined in the TEM images, the XRD analysis of the same sample produced a diffractogram typical of an amorphous material with some crystalline components (Figure 2H), with low intensity peaks overlapping on a featureless background. This was consistent with reports for Fe–B alloys in the literature,^14^ particularly in terms of a possible carbon contamination during LAL in organic solvents.^49,50^ Therefore, the crystalline structure of the Fe–B NPs was investigated at the single NP level using HRTEM, which confirmed the amorphous phase (Figure 2I) without any definite interplanar spacing. This was evident from the fast Fourier-transform (FFT) pattern, which showed no spots, unlike phases with crystalline order. Additionally, the HRTEM images revealed an organic layer surrounding the NPs (Figure 2I). EDX elemental analysis mapping was also performed (Figures 2J and S1 in Supporting Information (S.I.)), with the results indicating a homogeneous Fe distribution in the Fe–B NPs and the absence of an oxygen signal. Although the B signal was below the detection limit, the results nevertheless indicated that there was neither massive B segregation nor single B NPs present.

Using the Rietveld refinement, the low intensity reflections in the XRD diffractogram were identified as crystalline FeB (65 wt %), Fe_2_B (1 wt %), Fe (6 wt %), and B_2_O (28 wt %). Finally, ICP–MS analysis confirmed the presence of Fe and B in the Fe–B@PVP–PEG–ASA–CA sample, with an atomic ratio of 66% for Fe and 34% for B. These values indicated that the NP composition was substantially equivalent to the target (Fe/B 65/35 at%). Therefore, the NPs mainly comprised amorphous domains with a minor component of crystalline Fe–B alloy and B-doped Fe NPs, which presumably included boron oxide at the particle surfaces as a result of B oxidation with oxygen and water.^14,16,52,53^

### 3.2 Au–Fe NPs

The six Au–Fe NP samples were initially analyzed using UV–Vis–NIR spectroscopy (Figure 3A). Similar to the Fe–B NPs, the Au–Fe NPs had almost featureless spectra with no discernible absorption peak. However, an absorption edge was observed at ∼ 500 nm, attributed to the weak plasmonic behavior of these nanoalloys.^15^ Additionally, the optical extinction decreased until reaching almost zero at wavelengths >900 nm, unlike the Fe–B NPs. These features were common to all the Au–Fe NP samples; however, the absolute absorbance varied considerably, indicating a different yield when different polymeric coatings were used. As with the Fe–B NPs, the absorption spectrum profiles did not change with the type of ligand, indicating that the IA NP composition was unaffected by the polymer type.

**Figure 3:**
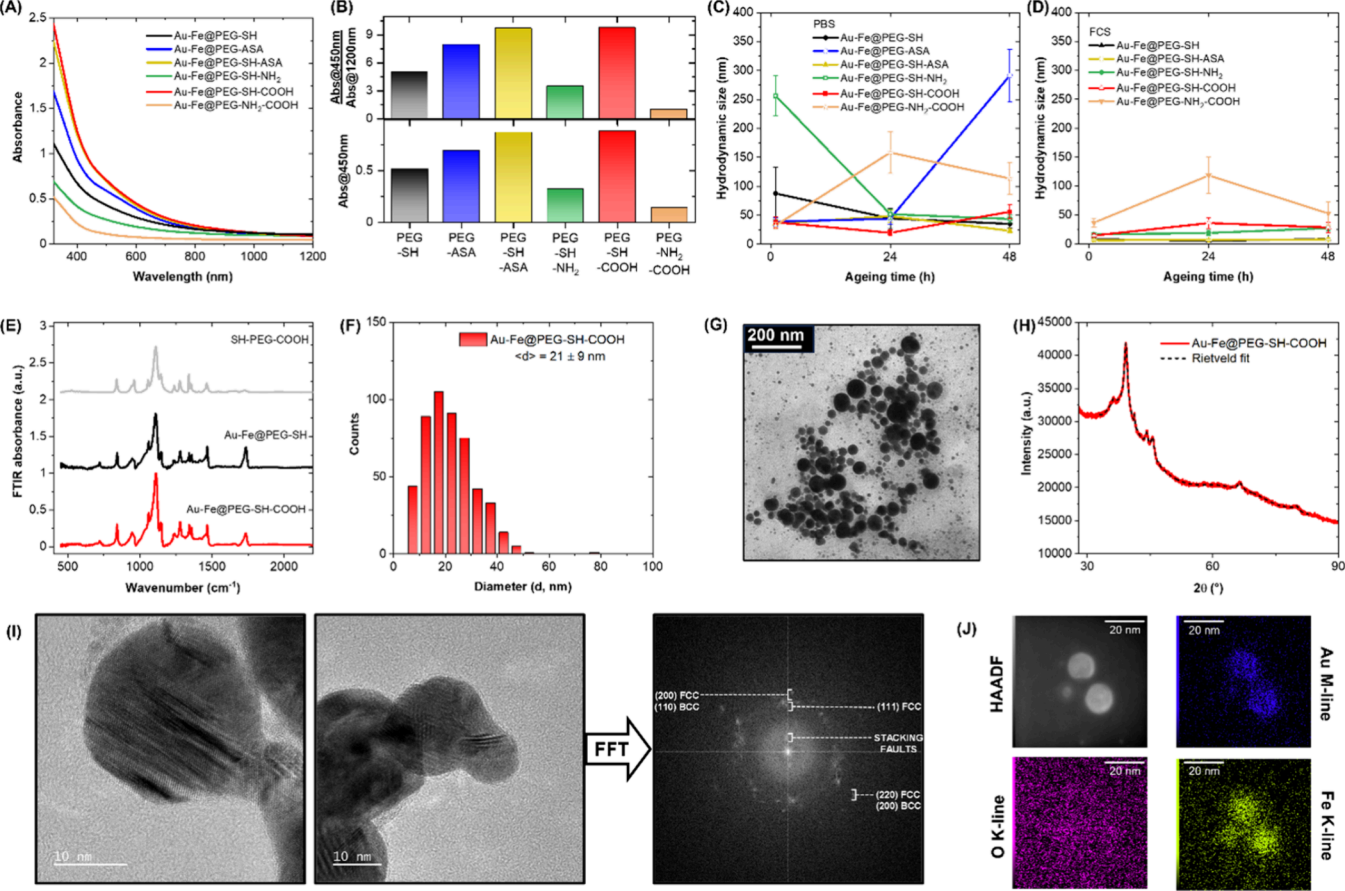
(A) UV–Vis–NIR spectra of the different Au–Fe NP formulations: PEG–SH (black), PEG–ASA (blue), PEG–SH–ASA (yellow), PEG–SH–NH_2_ (green), PEG–SH–COOH (red), and PEG–NH_2_–COOH (pink). (B) Bottom: absorbance@450 nm for the six samples, indicating their concentration and synthesis yield. Top: absorbance@450 nm/absorbance@1200 nm ratio, indicating the effective dispersion of the Fe–B NPs versus their aggregation among the samples. (C, D) Hydrodynamic size of the six Au–Fe NP formulations after aging from 1 to 48 h in PBS (C) and FCS (D). (E) FTIR spectra of Au–Fe@PEG–SH–COOH (red), Au–Fe@PEG–SH (black), and a reference SH–PEG–COOH powder (gray). (F, G) Au–Fe@PEG–SH–COOH size distribution obtained using TEM analysis of >500 NPs (F) and representative TEM image (G). (H) XRD diffractogram of Au–Fe@PEG–SH–COOH (red line) and Rietveld refinement (black dashed line). (I) HRTEM and related FFT of Au–Fe@PEG–SH–COOH NPs. (J) EDX mapping indicates the coexistence of Fe and Au in the Au–Fe NPs and the absence of an oxygen signal.

For the Fe–B NPs, absorbance at 450 nm was considered the end point for the synthesis yield, which was maximum for the Au–Fe@PEG–SH–ASA and Au–Fe@PEG–SH–COOH samples (Figure 3B, bottom). However, the absorbance@450 nm/absorbance@1200 nm ratio (Figure 3B, top) was indicative of NP stability and the absence of large agglomerates, which was also maximum for the Au–Fe@PEG–SH–ASA and Au–Fe@PEG–SH–COOH samples.

DLS measurements were performed in PBS (Figure 3C) and FCS (Figure 3D) to verify the hydrodynamic size of the six samples and assess the best coating procedure for optimal stability in these biologically relevant media. The hydrodynamic size in PBS remained stable for the Au–Fe@PEG–SH–ASA and Au–Fe@PEG–SH–COOH samples but increased in the Au–Fe@PEG–NH_2_–COOH and Au–Fe@PEG–ASA samples and decreased in the Au–Fe@PEG–SH–NH_2_ and Au–Fe@PEG–SH samples because of aggregation. The size decrease was attributed to the dissolution of Fe oxide components in this type of liquid environment, as previously observed.^15,54^ In FCS, the Au–Fe@PEG–ASA sample was unstable and could not be measured. The Au–Fe@PEG–NH_2_–COOH sample showed a consistent size increase, whereas the hydrodynamic size of the other samples remained more homogeneous and <50 nm for 48 h, indicating that these formulations were generally stable. The Au–Fe@PEG–SH and Au–Fe@PEG–SH–ASA samples had hydrodynamic sizes of <10 nm after 48 h (8 ± 1 nm and 8 ± 2 nm, respectively), which were lower than in PBS (35 ± 7 nm and 23 ± 4 nm, respectively, after 48 h). This suggests that the serum protein covered the NP surfaces and replaced the polymeric coating, initiating NP dissolution. The Au–Fe@PEG–SH–COOH sample exhibited different behavior, maintaining a similar size in both PBS (56 ± 12 nm) and FCS (29 ± 9 nm) even after 48 h. This indicates that the sample had better structural stability and thus a more homogeneous polymeric shell. Overall, the Au–Fe NPs functionalized with the mPEG–SH and SH–PEG–COOH mixture showed the best optical absorption and DLS results. Additionally, the z-potential analysis results of the Au–Fe@PEG–SH–COOH and reference^15^ Au–Fe@PEG–SH samples in PBS were – 9 ± 1 mV and – 3.4 ± 0.4 mV, respectively. Although these z-potentials are not large enough to justify a purely electrostatic colloidal stabilization, the Au–Fe@PEG–SH–COOH sample had a higher value, which was consistent with the better colloidal stability indicated by the UV–vis–NIR and DLS analyses.

To evaluate the effective surface functionalization, the FTIR spectrum of the Au–Fe@PEG–SH–COOH sample was compared to that of the reference Au–Fe@PEG–SH sample and an SH–PEG–COOH powder. As stated above, the entire unbound polymer was removed during repeated dialysis cycles with pure water (Figure 1); therefore, the polymer detected using FTIR was bound to the NPs. The results (Figure 3E) clearly indicated the presence of the PEG chains in both NP formulations because of CH_2_ bending at 840 cm^–1^, C–C and CH_2_ rocking at 1010 cm^–1^, C–O–C stretching at 1100 cm^–1^, CH_2_ wagging and stretching at 1280 cm^–1^, C–H bending at 1370 cm^–1^, and the well-known alkyl C–H stretching at 2900 cm^–1^.^15,48^ Because the FTIR spectra of the SH–PEG–COOH and mPEG–SH were dominated by the 5 kDa PEG backbone, it was not possible to discriminate the presence of the – COOH groups. The Au–Fe@PEG–SH–COOH sample was analyzed further using TEM (Figure 3F and G) and XRD (Figure 3H). According to the TEM images, the Au–Fe NPs were spherical, which is typical for LAL synthesis, with an average size of 21 ± 9 nm. The XRD pattern displayed relatively large peaks, indicating a small crystallite size and high defectivity for these IA NPs. The Rietveld analysis performed on the XRD pattern identified the Au–Fe alloy phases with face-centered cubic (FCC) and body-centered cubic (BCC) symmetry. The FCC Au–Fe phase has a refined lattice parameter of 3.97780 ± 0.00025 Å, which is less than that of pure Au (4.079 Å) because of the presence of substitutional Fe atoms in the FCC lattice.^15,54^ Similarly, the BCC Au–Fe phase has a refined lattice parameter of 2.89853 ± 0.00068 Å, which is larger than that of pure Fe (2.8665 Å) because of the presence of substitutional Au atoms.^54^ The crystalline structure of the Au–Fe NPs was investigated at the single NP level using HRTEM (Figure 3I), which revealed a highly defective crystalline structure with several planar dislocations and stacking faults, some of which had a specific periodicity. This was confirmed by the FFT pattern, which showed several spots distributed around the reflections of the FCC and BCC phases, as well as some spots with large periodicity generated by the stacking faults observed in the HRTEM images. Additionally, the organic coating surrounding the NPs was evident in the HRTEM images (Figure 3I).

EDX elemental analysis mapping was also performed (Figures 3J and S2 in S.I.), with the results indicating the coexistence of Au and Fe in the Au–Fe NPs and the absence of an oxygen signal. ICP–MS analysis confirmed the presence of Fe and Au in the Au–Fe@PEG–SH–COOH sample, with atomic ratios of 67% for Fe and 33% for Au. These values indicated that the NP composition was close to the target (Au/Fe 25/75 at%), although some Fe was lost during the LAL, consistent with previous observations.

### 3.3 MRI and Photothermal Performances

Both Fe–B and Au–Fe alloy NPs are known for their ability to shorten the transverse relaxation time (T_2_) of protons in nearby water molecules, thus acting as negative contrast agents for MRI.^14,15,54^

The MRI performance of the IA NPs in this study was also investigated, with different concentrations properly distributed in a series of agarose phantoms, using the described MRI tomograph operating at preclinical (7.0 T) field and an NMR system operating at clinical (1.5 T) magnetic field (Figure 4). At 1.5 T, the r_2_ values of the Fe–B (46 ± 4 mM_Fe^–1^ s^–1^) and Au–Fe (44 ± 2 mM_Fe^–1^ s^–1^) NPs were equivalent within the error (Figure 4A). However, at 7 T, the r_2_ value of the Fe–B NPs (101 ± 5 mM_Fe^–1^ s^–1^) was greater than that of the Au–Fe NPs (62 ± 3 mM_Fe^–1^ s^–1^) (Figure 4B). The contrast enhancement as a function of NP concentration was also evident from the MRI images of phantom cross sections collected at 7 T (Figure 4C).

**Figure 4:**
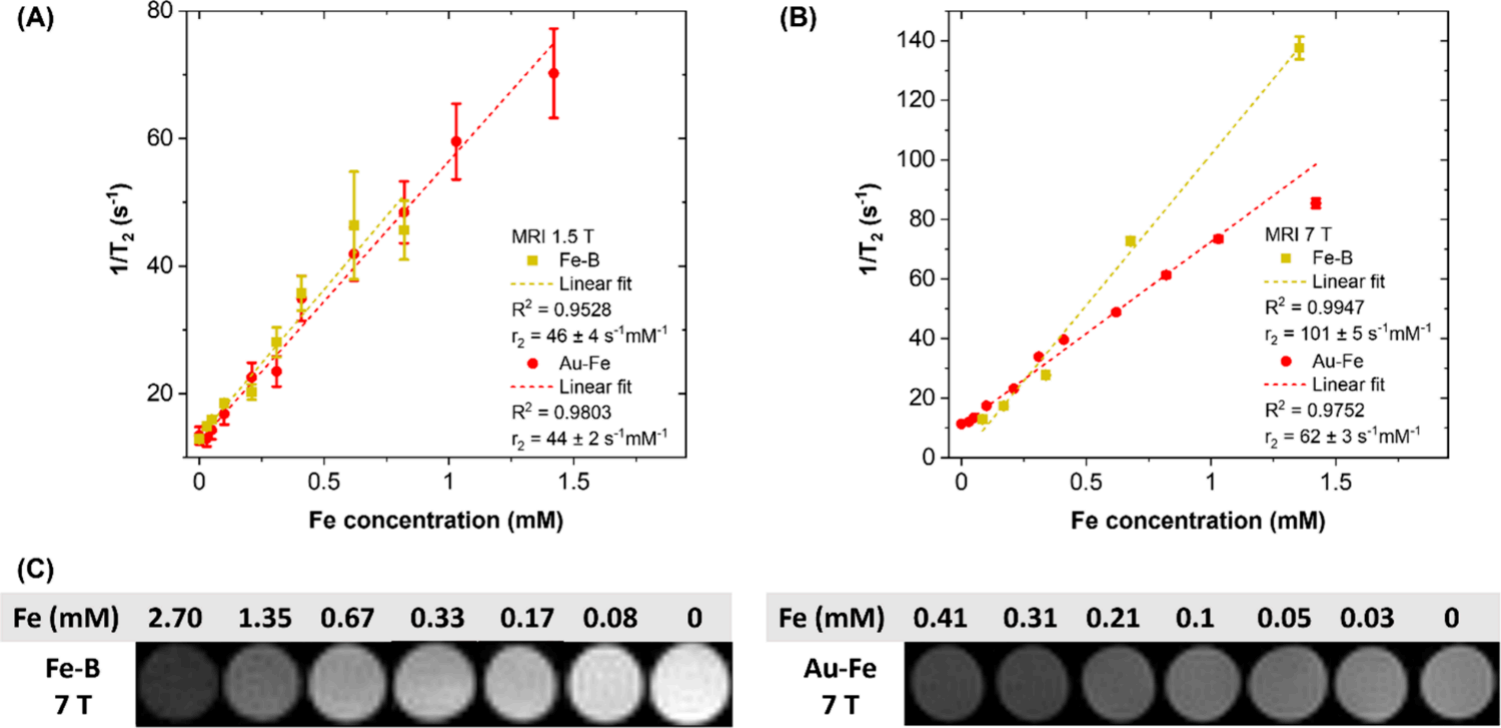
Transversal relaxation rate (1/T_2_) versus Fe concentration collected on phantoms in agarose gel containing the Fe–B@PVP–PEG–ASA–CA (yellow dots) and Au–Fe@PEG–SH–COOH (red dots) samples at various dilutions collected at clinical (1.5 T, A) and preclinical (7 T, B) fields. Dashed lines are the linear fit of experimental data, providing the corresponding relaxivity for each condition tested. (C) MRI images of phantom cross sections collected at 7 T, showing a negative contrast increasing with Fe concentration. The different contrast in the 0 mM_Fe phantoms of the Fe–B and Au–Fe samples is due to the use of differences in the acquisition parameters (see Materials and Methods section).

The photothermal properties of the Fe–B@PVP–PEG–ASA–CA and Au–Fe@PEG–SH–COOH formulations were evaluated under NIR light (785 nm) after dispersing the samples in 1 wt % agarose phantoms at a 0.091 ± 0.009 mg_Fe/mL concentration. Different laser powers were applied, ranging from 0.374 to 0.534 W, and the temperature reached by the phantoms was monitored over time. The temperature plateaus were observed after 10^3^ s, with the maximum temperature scaling with the incident laser fluence in both the IA NPs tested (Figure 5A and B). As shown in Figure 5C, temperature increased linearly as laser power increased, with the Fe–B NPs achieving the highest temperatures at parity with incident laser power. The thermographic images indicated a maximum temperature of 75.4 °C ± 1.0 °C for Fe–B@PVP–PEG–ASA–CA and 55.3 °C ± 1.0 °C for Au–Fe@PEG–SH–COOH under 0.534 W irradiation (insets of Figure 5A and B). The linearity in Figure 5C, as well as the stability of the plateaus in Figure 5A and B, indicated that the IA NPs were photostable. The temperature increase was especially appreciable for the Fe–B NPs, considering their low mass concentration, resulting in values comparable to or higher than those reported for several plasmonic nanostructures based on noble metals or calchogenides.^55,56^

**Figure 5:**
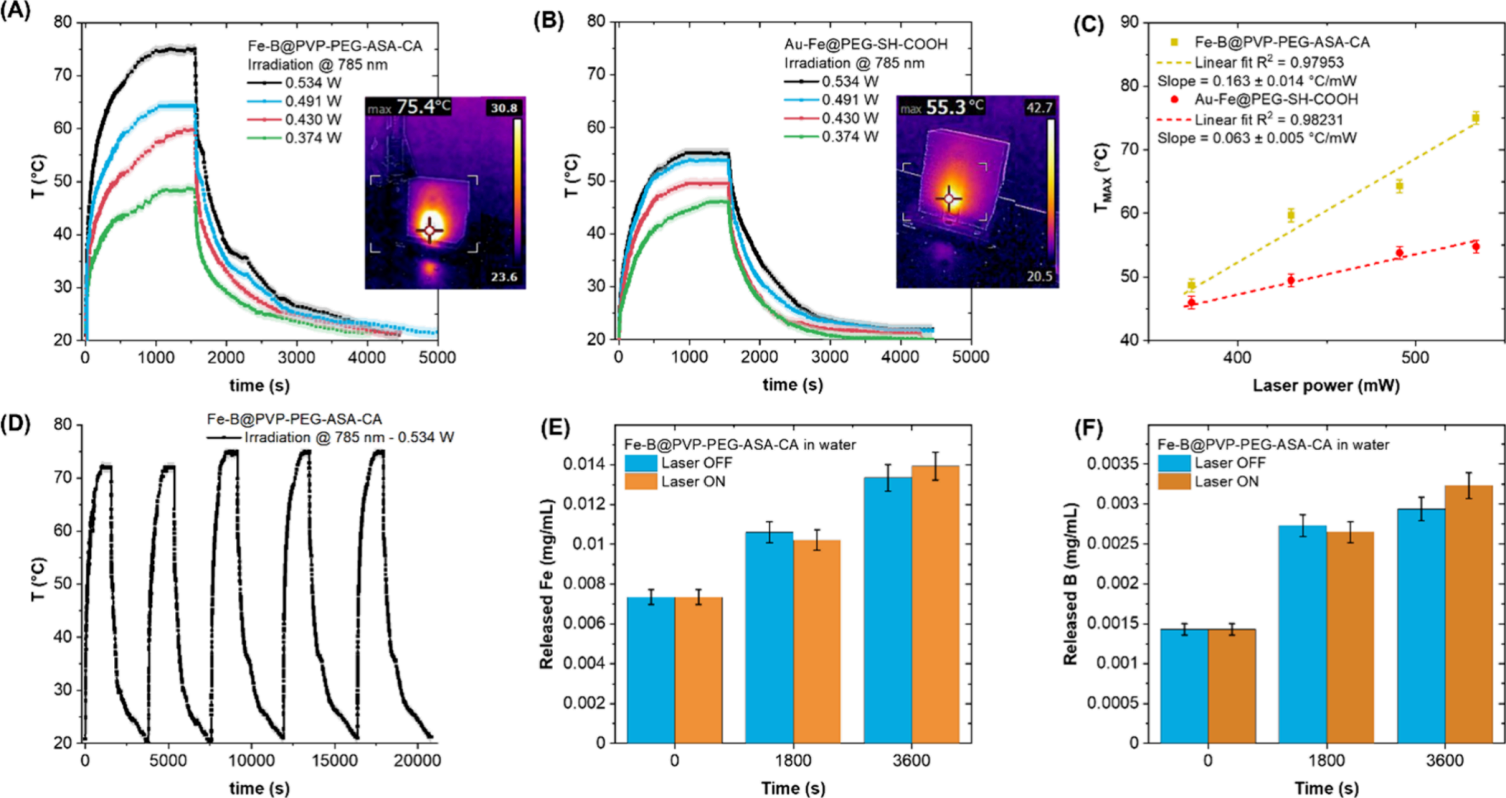
(A, B) Plot of temperature as a function of time in a phantom of Fe–B@PVP–PEG–ASA–CA (A) and Au–Fe@PEG–SH–COOH (B) irradiated with a cw 785 nm laser source at different powers. The shadowed area indicates the measurement tolerance of ± 1 °C. Insets show the thermographic images of phantoms at the temperature plateau under 0.534 W irradiation. (C) Plot of the plateau temperatures in the two samples as a function of laser power. Dashed lines are the linear fits, indicating the better performance of the Fe–B NPs and the photostability of the two types of IA NPs. (D) Heating/cooling cycles at 0.534 W for the sample, indicating its photostability. (E, F) Concentration of Fe (E) and B (F) in distilled water after 0, 1800, and 3600 s exposure to Fe–B@PVP–PEG–ASA–CA NPs in the dark (blue histograms) or under 0.534 W laser illumination (orange histograms), indicating that no NP photodegradation occurred.

The thermal stability of the best performing NPs (Fe–B@PVP–PEG–ASA–CA) was tested under five repeated laser irradiation cycles at 534 mW (Figure 5D), which showed that the sample was not photodegradable. Additionally, the release of Fe and B in water was measured in the dark or under 534 mW laser irradiation at different time points. The results indicated that photothermal heating was not associated with increased NP dissolution and metal ion release in the liquid solution (Figure 5E and F).

### 3.4 *In* *Vitro* and *In* *Vivo* Tests of Fe–B NPs

Because the Fe–B NPs showed the best photothermal performance, their exploitability for photothermal treatment of cancer cells was investigated in preliminary *in vitro* experiments. *In vitro* cytotoxicity assays revealed that the Fe–B NPs were safe up to 50 μg_Fe/mL, even after 48 h (Figure 6A). To further evaluate the general biocompatibility of Fe–B NPs, cytotoxicity assays were performed on WI-26 human lung fibroblasts, a normal cell line. The results indicated that Fe–B NPs were well tolerated by these normal human cell lines up to 100 μg_Fe/mL after 24 and 48 h (Figure S3 in S.I.). To confirm Fe–B NP internalization, PB staining was performed after 24 and 48 h of incubation with 50 μg_Fe/mL of NPs (Figure 6B). The images showed some degree of NP aggregation in the proximity of the cells after 24 h, likely contributing to the toxicity observed at concentrations >50 μg_Fe/mL. After 48 h, fewer extracellular clusters were visible, with an increased presence of internalized Fe–B NPs within the cells. The ultrastructural cell morphology (Figure 6C) confirmed the presence of Fe–B NPs within endocytic vesicles in the cytoplasm after 24 h of incubation. Notably, a higher degree of Fe–B NP internalization was observed after 48 h, with Fe–B NPs localized not only within endocytic vesicles but also inside organelles (mitochondria and nuclei).

**Figure 6:**
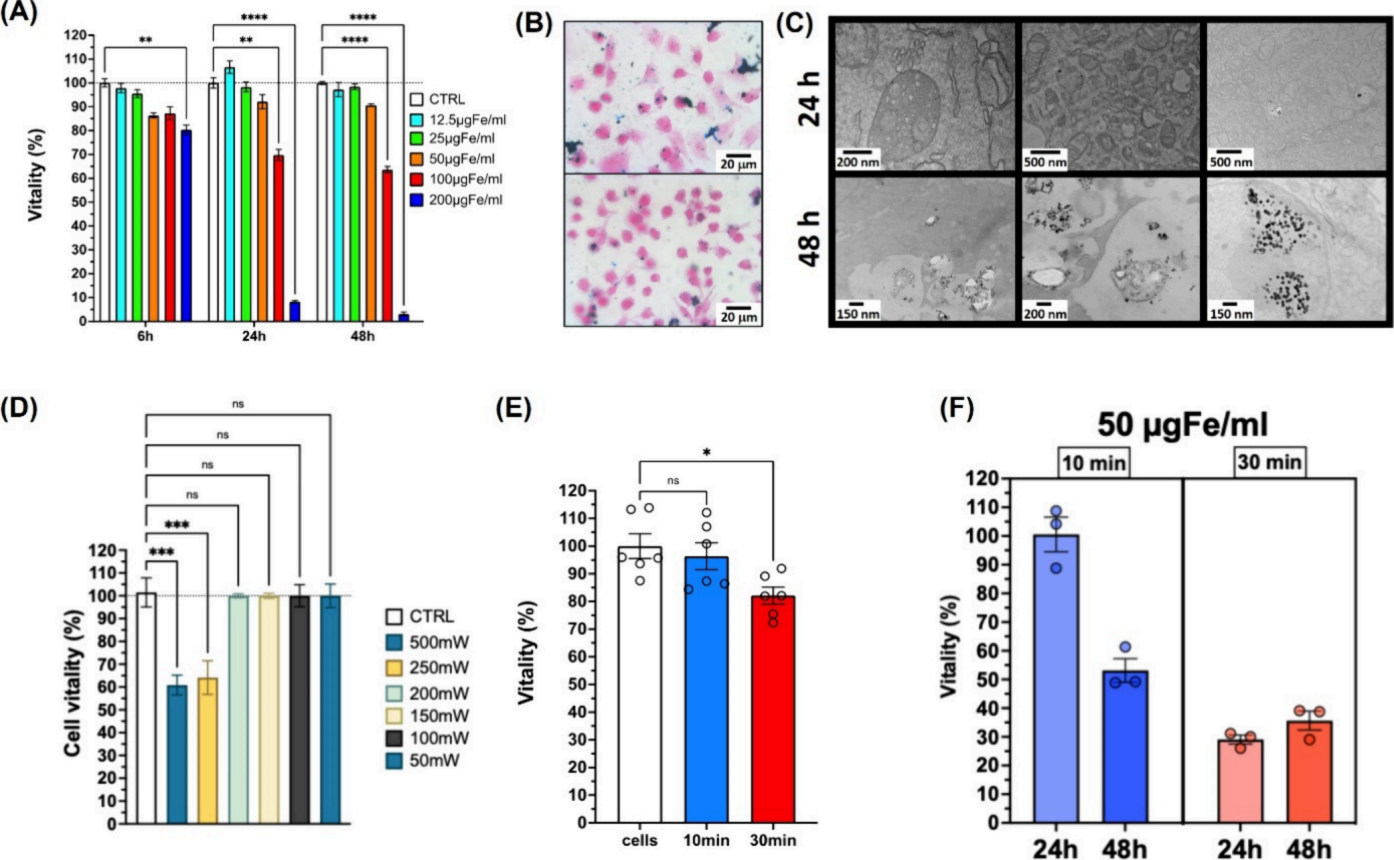
(A) Cytocompatibility determined using MTT of Fe–B NPs incubated with A375 cells at various concentrations (12.5–200 μg_Fe/mL) for 6, 24, and 48 h. (B, C) Assessment of cell uptake after 24 and 48 h incubation of the Fe–B NPs, using optical microscopy in PB-stained cells (B) and TEM imaging (C). (D) Effect of 785 nm laser irradiation at various powers on the vitality of cells without NPs, indicating that negligible effects are obtained below 200 mW for a treatment time of 10 min. The vitality was assessed at 24 h after irradiation treatment. (E) A375 cell vitality after 200 mW laser irradiation at 785 nm for 10 or 30 min. The vitality was assessed at 24 h after irradiation treatment. (F) Cell vitality after 200 mW laser irradiation at 785 nm for 10 or 30 min of A375 cells incubated with the Fe–B NPs at 50 g/mL for 24 or 48 h. The vitality was assessed at 24 h after irradiation treatment. (ns = not significant; * *p* < 0.05; ** *p* < 0.01; *** *p* < 0.001; **** *p* < 0.0001).

Then, the safety (Figure 6D and E) and efficacy (Figure 6F) of the photothermal treatment were investigated. Initially, the change in cell viability at 24 h after laser irradiation for 10 min at various laser powers (500, 250, 200, 150, 100, and 50 mW) was tested. As shown in Figure 6D, cell viability decreased to ∼ 60% at the highest powers (500 and 250 mW), whereas below 200 mW, the vitality was not influenced. When the irradiation time was increased to 30 min at 200 W (Figure 6E), the vitality at 24 h after irradiation was still >80%. Under these conditions (200 W and irradiation times of 10 and 30 min), A375 cells incubated with the Fe–B NPs (24 h, 50 μg_Fe/mL) exhibited a statistically significant reduction in cell vitality at 24 h after irradiation treatment for 30 min (29% ± 3%, *p* < 0.0001). When the cells were incubated with NPs for 48 h, the vitality at 24 h after irradiation was significantly lower (53% ± 4% after 10 min of irradiation, *p* < 0.0001; 36% ± 3% after 30 min, *p* < 0.001, Figure 6F). Instead, no statistically significant difference was observed between cells irradiated for 30 min, regardless of whether they had been incubated with Fe–B NPs for 24 or 48 h (Figure 6F).

Finally, the biodistribution of the Fe–B@PVP–PEG–ASA–CA NPs was assessed *in vivo* via longitudinal MRI analysis in the liver, spleen, and kidneys of nude mice (Figure 7). To enable a more accurate comparison across different time points, signal intensities (SI) were normalized against a reference water tube and expressed relative to it, e.g., SI_organ/SI_standard (Figure 7A). Postinjection, a reduction in normalized SI was measured in the liver, spleen, and kidneys, consistent with NP accumulation and distribution in the bloodstream, with the associated shortening of the transversal relaxation time. In the liver, the normalized signal intensity decreased considerably at 1 h postinjection (0.037 ± 0.005) compared to the preinjection value (0.094 ± 0.010), and remained suppressed at 72 h (0.041 ± 0.009). Similarly, the spleen showed a decrease from 0.092 ± 0.052 (pre) to 0.048 ± 0.022 (24 h), with partial recovery by 72 h (0.056 ± 0.024). In contrast, the kidney signal intensity showed a lower fluctuation from 0.338 ± 0.020 (pre) to 0.310 ± 0.027 (after 5 h), until recovery to 0.362 ± 0.009 (72 h), suggesting limited renal accumulation compared to the liver and spleen. These results confirmed effective *in vivo* tracking of the Fe–B NPs via MRI and demonstrated predominant accumulation in the liver and spleen, as supported by the T_2_-weighted images (Figures 7B and S4 in S.I.), indicating reticuloendothelial system-mediated clearance.^14,15^

**Figure 7:**
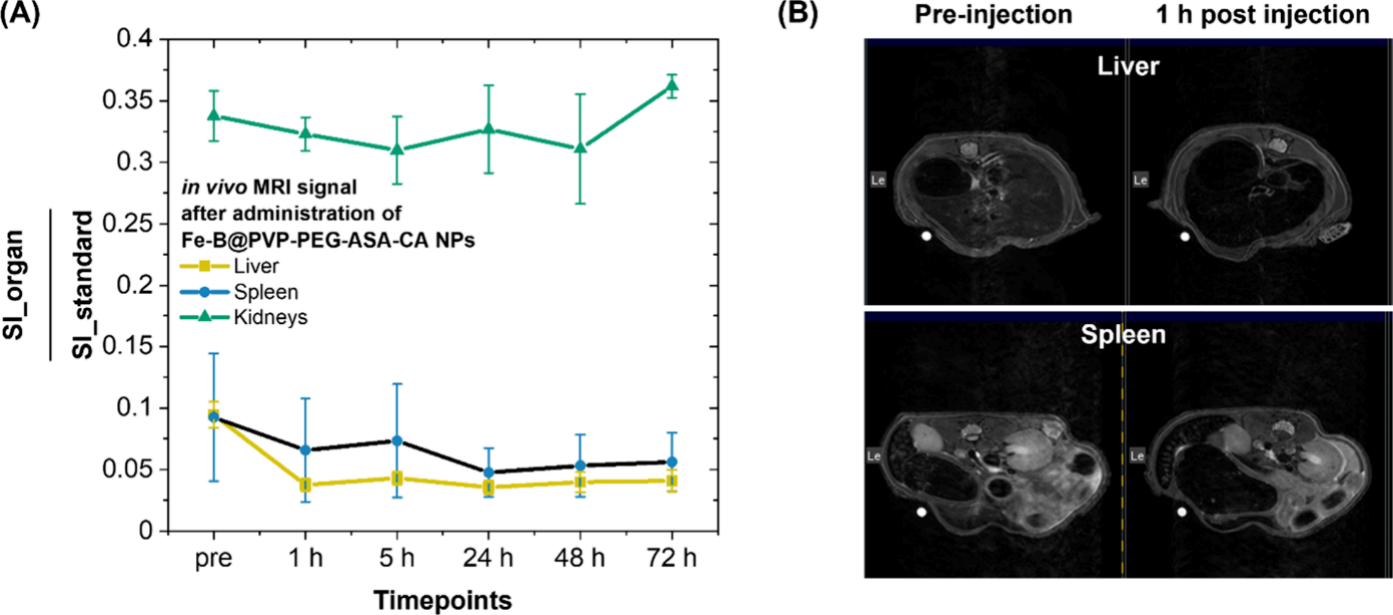
(A) Relative signal intensity (SI) measured in the liver, spleen, and kidneys of nude mice after administering Fe–B@PVP–PEG–ASA–CA NPs at various time points (*N* = 3; data reported as mean ± SD). Fe–B NPs were administered intravenously via tail vein injection at a dose of 4.0 mg_Fe/kg. (B) Representative T_2_-weighted MRI images of the liver and spleen in nude mice models before and 1 h after administration of the Fe–B@PVP–PEG–ASA–CA NPs. A standard color bar for all images was used.

Histological examination combining PB and H&E staining was performed to evaluate both Fe accumulation and tissue integrity after intravenous administration of the Fe–B NPs. As shown in Figure S5 (S.I.), all analyzed organs (kidney, heart, spleen, liver, and lung) from NP-treated mice displayed normal histological architecture, comparable to the controls. In PB-stained sections, Fe deposits appeared as discrete blue signals, indicating the presence of Fe–B NPs mainly in the liver and spleen, with fewer deposits in the kidney, heart, and lung. These localized signals corresponded to the physiological sequestration of Fe-containing particles by macrophage populations, such as Kupffer cells in the liver and splenic macrophages in the red pulp. H&E counterstaining confirmed a preserved and normal histoarchitecture in all tissues. Hepatocytes, renal tubules, cardiac fibers, and alveolar structures appeared intact, with no evidence of necrosis, inflammation, or fibrosis. Overall, the combined PB/H&E staining demonstrated that the Fe–B NPs underwent expected biodistribution, maintaining tissue integrity and causing no detectable histopathological damage.

### 3.5 Polymer Coating and Colloidal Stability

In this study, different polymeric coatings were selected according to the specific surface chemistry of the IA NPs to identify the best strategy for particle stabilization. Because Fe and B have low reduction potentials and are easily oxidized at NP surfaces, surface stabilization can be achieved by employing functional groups that can coordinate metal ions or undergo attractive physical interactions. According to the literature, this is the case with carboxylic groups in citrate ions and PEG–ASA,^57,58^ as well as the carbonyl group in PVP.^59^ Citrate ions also dissolve weakly crystalline Fe oxide and hydroxide compounds, clean IA NP surfaces, and facilitate polymer coordination.^14^ The best results were observed when PVP, PEG–ASA, and CA were used simultaneously, thus combining the advantages of the three compounds in terms of chemical composition and molecular weight (40 kDa for PVP, 20 kDa for PEG–ASA).

In the case of Au–Fe nanoalloys, the Au atoms at the NP surfaces considerably improves their stability by establishing Au–S chemical bonds with any polymer chain bearing a thiol or disulfide group. In fact, all the thiolated polymers provided better Au–Fe NP stabilization than the nonthiolated PEG–ASA, despite the latter having several carboxylic groups that can effectively interact with surface Fe atoms. However, the carboxylic groups provided an advantage when combined with thiol groups, with the Au–Fe@PEG–SH–COOH sample demonstrating the best colloidal stability. Unlike the methyl (Au–Fe@PEG–SH) and amine (Au–Fe@PEG–SH–NH_2_) groups, the carboxylic group is acidic and, at physiological pH (7.4), contributes to electrostatic stabilization in addition to the steric stabilization already provided by the PEG chain. The carboxylic groups in the samples with PEG–ASA (Au–Fe@PEG–ASA and Au–Fe@PEG–SH–ASA) were not effective for improving the colloidal stability of the NP, contrary to Au–Fe@PEG–SH–COOH, indicating that PEG–ASA did not effectively bind to the NPs because of the lack of thiol groups.

The ensemble (XRD) and single particle (HRTEM) analyses showed the complex atomic structure of the Fe–B (amorphous) and Au–Fe (highly defective) NPs. LAL is an indispensable methodology for synthesizing such metastable amorphous or crystalline phases, which are dispersed as a colloid rather than agglomerated as a powder. Most importantly for this study, using LAL, the NPs can be coated *in situ* with different ligands to screen the best formulation for biomedical applications. These key advantages complement the general features of the LAL synthesis, which include its simplicity, operation at room temperature and pressure, low cost, low environmental impact, and the ability to use only bulk metal components and common solvents (e.g., ethanol, acetone, or, in several cases, water containing biocompatible polymers or other nontoxic compounds such as citrate).^34–38^ Notably, all of this comes at the expense of consistent particle polydispersity, which may be mitigated after LAL using SBS.^40^ This was shown for the most relevant formulation (Fe–B@PVP–PEG–ASA–CA NPs), indicating that a refinement of the size distribution was possible using this technique (Figure S6 in S.I.).

The IA NPs with the best stability and synthesis yield were also tested against their functional properties, such as contrast generation in MRI and photothermal heating with NIR light. It is well-known that Fe-based compounds are exploitable for MRI because they induce local magnetic field inhomogeneities, which affect the relaxation times of surrounding protons in tissue.^3,5–7^ Specifically, both Fe–B and Au–Fe NPs are known to act as contrast agents for T_2_-weighted MRI. Stability is essential for maintaining a linear and intense response during MRI measurements, preventing NP agglomeration and precipitation under the intense magnetic fields used in clinical and preclinical tomographs. Additionally, the stability of the MRI contrast agent is required for the most advanced magnetic resonance approaches, such as dynamic monitoring and quantification of biomarkers and other elusive compounds (e.g., nitric oxide, hydrogen peroxide, and hydrogen sulfide) *in vivo*.^60–62^ In this case, good linearity was observed for the Fe–B@PVP–PEG–ASA–CA and Au–Fe@PEG–SH–COOH samples, both exhibiting an r_2_ comparable to that of the benchmark Fe oxide contrast agent for clinical use. The r_2_ values of the IA NPs were comparable to the values reported in the literature for clinically used Fe oxide NPs, which at 1.5 T can range from 20–188 ^63,64^ or 20–100 mM_Fe^–1^ s^–1^ ^65–67^ and <100 mM_Fe^–1^ s^–1^ at 7 T.^68^ Additionally, the Fe–B NP formulation remained stable in the fridge for up to 4 weeks after synthesis, whereas a dried deposit of the Au–Fe formulation was produced at the end of the synthetic procedure that could be stored in the fridge and resuspended in water or PBS immediately before use. The colloidal stabilities of the two formulations were tested using DLS after aging for 1 week in various environments (PBS, FCS, and plasma, Figure S7 in S.I.). The results indicated that the IA NPs maintained colloidal stability in PBS and FCS. The two types of IA NPs initially had a larger hydrodynamic size in plasma than in PBS and FCS, particularly the Au–Fe NPs, but after 1 week, their size decreased to levels comparable to the other environments. This change was consistent with the beginning of NP biodegradation, as described in previous studies. Additionally, the *in vivo* experiments confirmed the biodistribution of Fe–B NPs in the bloodstream before they accumulated in organs responsible for NP clearance, such as the liver and spleen.

### 3.6 Optical Properties

Owing to their challenging synthesis and metastability (the amorphous nature of Fe–B and the immiscibility of the Au–Fe alloy), many properties of these Fe nanoalloys remain unknown. For instance, the photothermal properties of the Fe–B and Au–Fe nanoalloys have not previously been investigated, although their optical spectra show consistent absorption in the NIR region, where tissues are more transparent to electromagnetic radiation, allowing the use of various theranostic approaches based on light stimulation.^10^ One of these approaches, which greatly benefits from the use of nanomaterials, is PTT.^10–12^ Hence, both Fe–B and Au–Fe compounds demonstrated the ability to convert light into heat by absorbing 785 nm radiation, with the B alloy showing the best performance at Fe concentration parity. In fact, Au is a well-known plasmonic material that exhibits a plasmon absorption in the visible region when the particle shape is spherical, both in pure form and in its alloys with transition metals.^69,70^ In transition metal alloys, single-electron interband transitions are a common source of light absorption, which occurs throughout a wide spectral range because of several occupied and unoccupied electronic levels around the Fermi energy. The optical properties of the two IA NPs can be better understood by comparing their mass absorption coefficient (ε_mass_, cm^–1^ mg^–1^ mL, Figure 8A) with that of benchmark spherical Au NPs (coated with PEG–SH, average size of 15 ± 9 nm) using the Lambert–Beer relation between absorbance (Abs), mass concentration (c, in mg/mL), and optical path (b, in cm) at a given wavelength (λ):

$$\mathrm{Abs}(\lambda )=\varepsilon_{\mathrm{mass}}(\lambda )\times b\times c$$ 1

**Figure 8:**
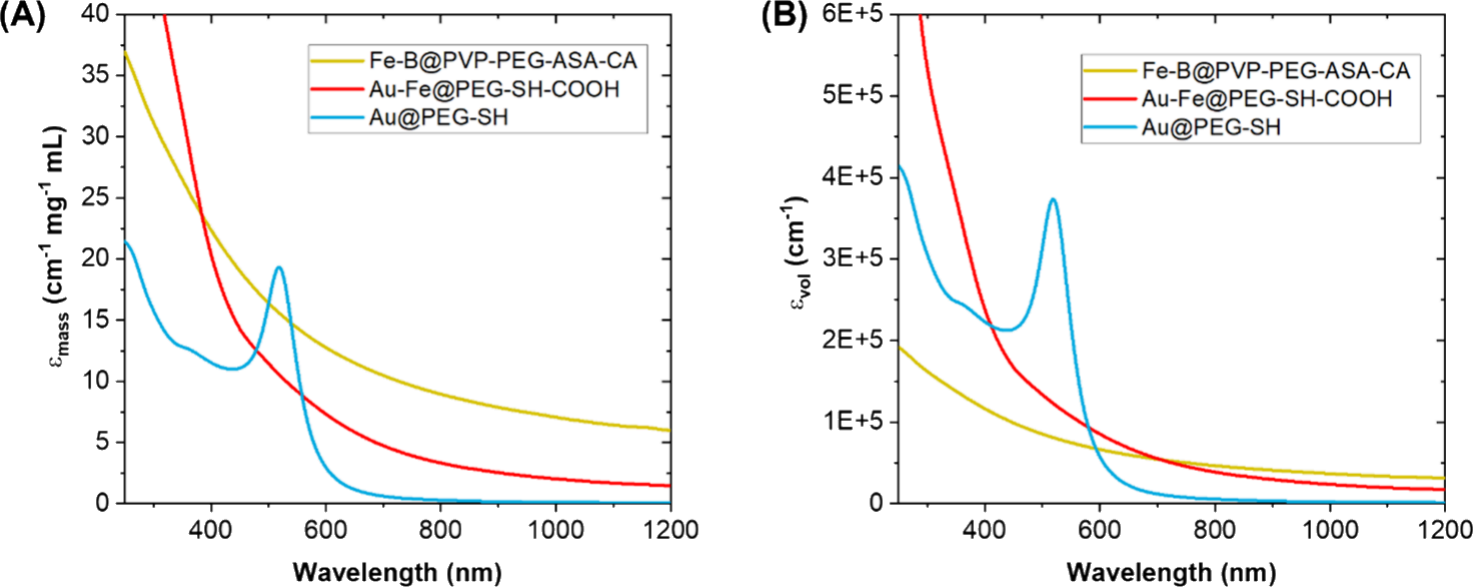
Plot of the mass absorption coefficient (ε_mass_, A) and the volume fraction absorption coefficient (ε_vol_, B) for the Fe–B (yellow lines) and Au–Fe (red lines) NPs, compared to a reference sample of PEG-coated Au NPs (blue lines).

The Fe–B NPs have a significantly larger ε_mass_ than Au NPs in the red and NIR spectral region. The Au–Fe NPs also have a larger ε_mass_ in the red and NIR region than spherical Au NPs. This is a clear advantage for any photothermal application requiring lightweight devices and earth-abundant, low-cost elements. The IA NPs have a superior ε_mass_ because of their favorable electronic structure for red and NIR light absorption, as well as the presence of light elements such as B and/or Fe, rather than high Z elements such as Au. To compare the optical absorption performance at the same NP volume, the volume fraction absorption coefficient (ε_vol_, cm^–1^, Figure 8B) should be considered according to the following equations:

$$\varepsilon_{\mathrm{vol}}(\lambda )=\varepsilon_{\mathrm{mass}}(\lambda )\times \rho$$ 2

$$\mathrm{Abs}(\lambda )=\varepsilon_{\mathrm{vol}}(\lambda )\times b\times f$$ 3

where ρ is the density of the material (mg/cm^3^), and f is the volume fraction of the material in the sample. For eq 3, it was assumed that 1 cm^3^ = 1 mL in the aqueous solution, and the density of IA NPs was obtained as a first approximation from the density of pure elements weighted on the alloy elemental composition. Under these assumptions, the superiority of Au NPs in the visible range is more evident because of their exceptionally large single particle absorption cross section.^71^ However, the volume-normalized absorption of IA NPs shows their superiority in the red and NIR region where the absorbance of Au nanospheres is negligible. The IA NPs also showed excellent photostability, which is crucial for long-term and repeated treatments.

Because the optical properties of the NPs scale with their volume,^71^ large NPs or NP agglomerates, such as those localized inside the cells in Figure 4B and C, have better photothermal effects at the nanoscale compared to isolated NPs. Nevertheless, it is well-known that plasmon absorption per unit volume in Au NPs depends slightly on particle size but dramatically on particle shape, with a red shift of the plasmon band when the symmetry of the NP shape is reduced (e.g., in a nanorod).^71^ However, unlike Au nanospheres or other Au nanostructures, Fe–B and Au–Fe NPs do not exhibit a sharp plasmon peak, suggesting that particle size and shape have a minor effect on optical absorption per unit volume.^71^ Therefore, although LAL does not allow control over particle size and morphology, this is unlikely to affect the optical properties of the spherical Fe–B and Au–Fe NPs. Notably, although Fe–B NPs have superior absorption at 785 nm, leading to better photothermal performance than Au–Fe NPs, elongated Au–Fe NPs with a composition of 33–67 at% can have a plasmonic absorption contribution in the NIR.^72,73^ However, these nanostructures have not yet been synthesized, thus their optical properties have not been compared to Fe–B and Au NPs with similar morphology.

The combination of MRI and photothermal heating in the NIR is novel for IA NPs and is of great interest for advanced theranostic applications, such as photosensitizer localization and quantification,^12,74–77^ the guidance of laser excitation sources or optical fiber probes in tumors,^12,75–77^ and the ability to measure local temperature in real time during treatment.^74,76,77^ Compared to clinically used photothermal agents, which are based on organic compounds,^55,56,78,79^ IA NPs have longer biopersistence and biodistribution times (Figure 7), form clusters inside the cells (Figure 6C), and offer the opportunity of *in vivo* tracking via MRI. Precise localization and quantification of photosensitizers are essential to ensure optimal accumulation in the targeted tumor region, maximizing therapeutic efficacy while minimizing potential toxicity and undesired effects in surrounding healthy tissues.^10–12,10–12,74–77^ Accurate guidance of the laser excitation source or optical fiber probes within the tumor is crucial for precisely directing photothermal treatment to the malignant tissues, improving therapeutic outcomes while reducing the risk of collateral damage to adjacent normal tissues.^10,12,75–77^ Real-time monitoring of local temperature during treatment enables fine control of the heating process, ensuring that the temperature remains within the therapeutic window to effectively ablate cancer cells while preventing excessive heat that could cause unintended damage and inflammation to healthy structures.^74,76,77^ For instance, the experiments in phantoms showed that the Fe–B NPs could reach a temperature of 75 °C for laser irradiation at 1.33 W/cm^2^ (Figure 5A), which is excessive for *in vivo* applications,^10,12,74–77^ and real-time MRI temperature monitoring can guide the operator in selecting an appropriate laser intensity to prevent photothermal damage to healthy tissues.

## 4 Conclusions

Effective stabilization of IA NPs is crucial for taking full advantage of their diverse properties and exploiting them in nanomedicine. Here, the optimal polymeric coatings for Fe–B and Au–Fe alloy NPs were identified. Fe–B NPs benefited from coatings with carboxylic and carbonyl groups from citrate, PEG–ASA, and PVP, with the best results observed when the three stabilizers were combined. In Au–Fe nanoalloys, Au–S bonds with thiolated polymers significantly improved NP stability, reaching the best results when carboxylic groups were present on the terminal part of the PEG–SH chain. Aside from stability, IA NPs demonstrated good MRI contrast agent ability and signal linearity in both clinical (1.5 T) and preclinical (7 T) fields. For the first time, photothermal capabilities were evaluated in the NIR region, where biological tissues are transparent. Fe–B@PVP–PEG–ASA–CA NPs provided excellent photothermal performance, outperforming the Au–Fe@PEG–SH–COOH NPs at the same Fe concentration. Additionally, IA NPs exhibited excellent photostability, supporting their long-term use. *In vitro* tests confirmed the biocompatibility and phototherapeutic potential of the Fe–B formulation. The dual MRI and NIR photothermal heating functionalities are of great relevance for precise imaging, laser guidance, and real-time temperature monitoring, enhancing PTT efficacy while minimizing collateral damage. These findings position IA NPs as a promising platform for integrated cancer imaging and treatment, deserving further validation with *in vivo* models in the future.

## Supplementary Material



## Notes

EDX elemental mapping of Fe–B and Au–Fe NPs; cytotoxicity of Fe–B NPs on WI-26 human lung fibroblasts; T_2_-weighted *in vivo* MRI images; histological evaluation; size refinement of Fe–B NPs; DLS of best Fe–Be and Au–Fe NP formulations after aging for 1 week (PDF)
