# Supplementary material for: Polymer Coated Iron–Boron and Gold–Iron Alloy Nanoparticles for Magnetic Resonance Imaging and Near Infrared Photothermal Applications
Source: ACS Appl Nano Mater. 2025 Dec 11;8(51):24512–27. doi: 10.1021/acsanm.5c04740 (PMC12752774; doi:10.1021/acsanm.5c04740)
Supplement: Supplementary file 1 [file an5c04740_si_001.pdf]

Supporting Information

Polymer Coated Iron-Boron and Gold-Iron Alloy Nanoparticles for Magnetic Resonance Imaging and Near Infrared Photothermal Applications

Michael Bissoli,[1,§] Alessandro Negri,[2,§] Asya Zerbato,[1] Denis Badocco,[1] Paolo Pastore,[1] Marta Filibian,[3,4] Francesca Brero,[5,4] Silvia Megalizzi,[5] Alessandro Lascialfari,[5,4] Nicola Greco,[2] Pasquina Marzola,[2] Vincenzo Amendola[1,*]

[1] Department of Chemical Sciences, University of Padova, 35131, Padova, Italy

[2] Department of Engineering for Innovation Medicine, University of Verona, Verona 37134, Italy

[3] Centro Grandi Strumenti, University of Pavia, 27100 Pavia, Italy

[4] National Institute for Nuclear Physics, Pavia Unit, Pavia 27100, Italy

[5] Department of Physics, University of Pavia, Pavia 27100, Italy

*email: vincenzo.amendola@unipd.it

[§] These authors contributed equally.

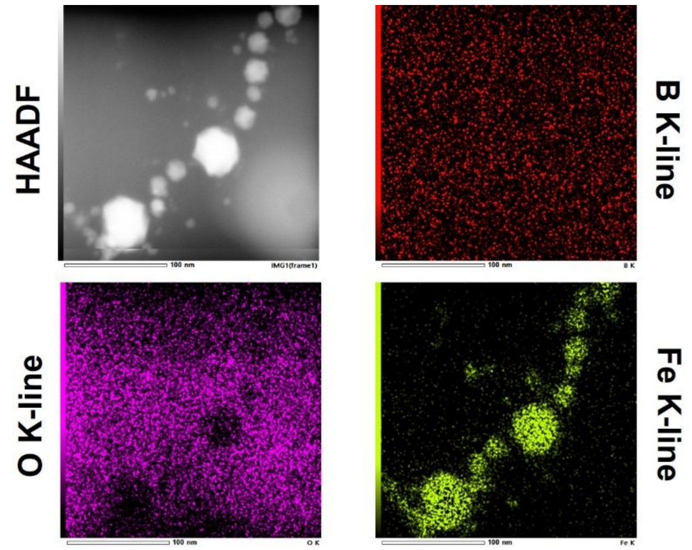

Figure S1. EDX mapping indicating homogeneous distribution of iron in the Fe-B NPs and absence of oxygen signal. The B signal is below the detection limit, thus indicating that no B segregation or single B NPs are present.

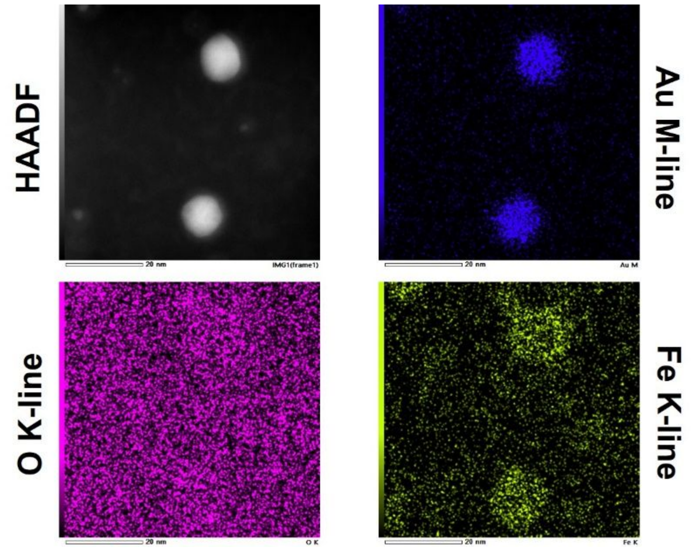

Figure S2. EDX mapping indicating the coexistence of iron and gold in the Au-Fe NPs and absence of oxygen signal.

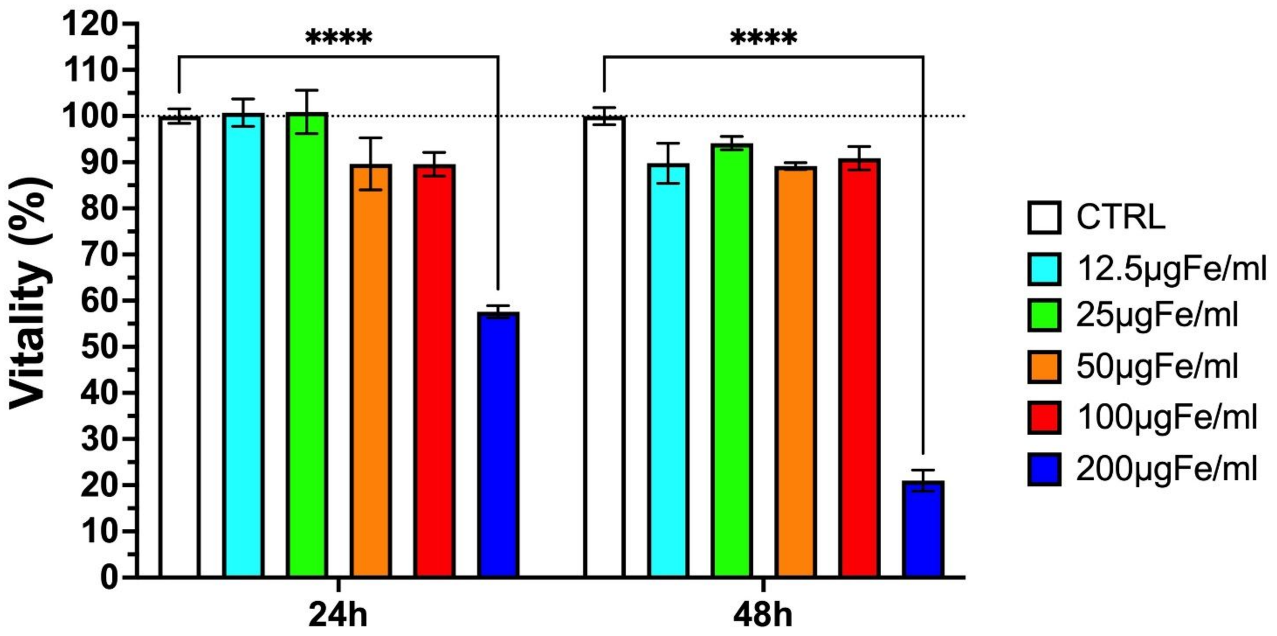

Figure S3. Cytocompatibility determined by MTT of Fe-B NPs incubated at various concentrations from 12.5 up to 200 µg_Fe/mL for 24 and 48 h with WI-26 human lung fibroblasts, a normal cell line. (ns = not significant; * $p < 0.05$; ** $p < 0.01$; *** $p < 0.001$; **** $p < 0.0001$).

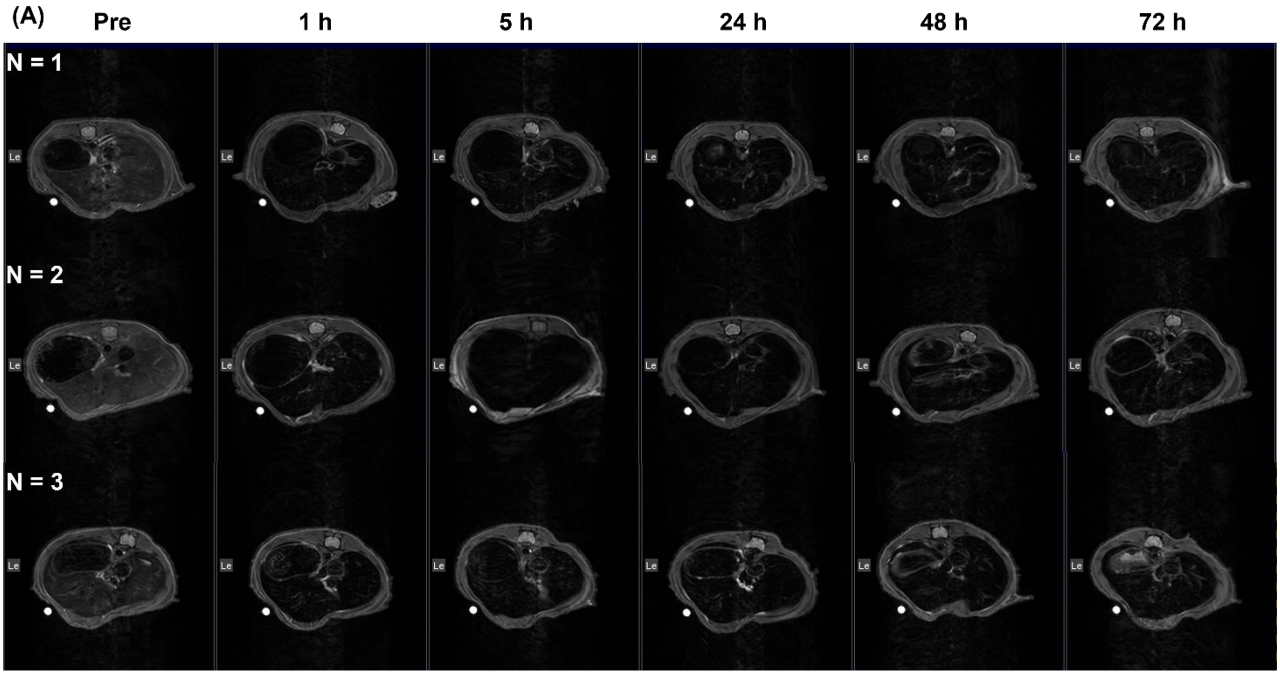

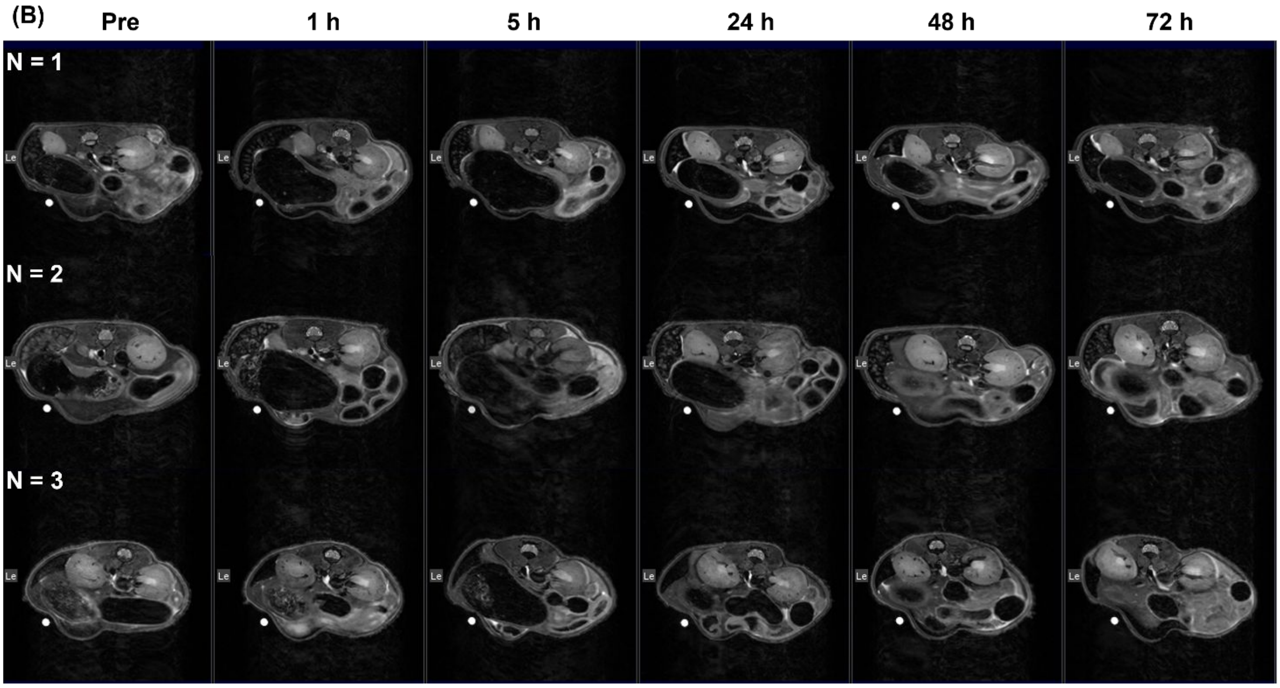

Figure S4. T_2-weighted MRI images of liver (A) and spleen and kidneys (B) of nude mice models before and after administration of the Fe-B@PVP-PEG-ASA-CA NPs. A standard color bar for all images was used.

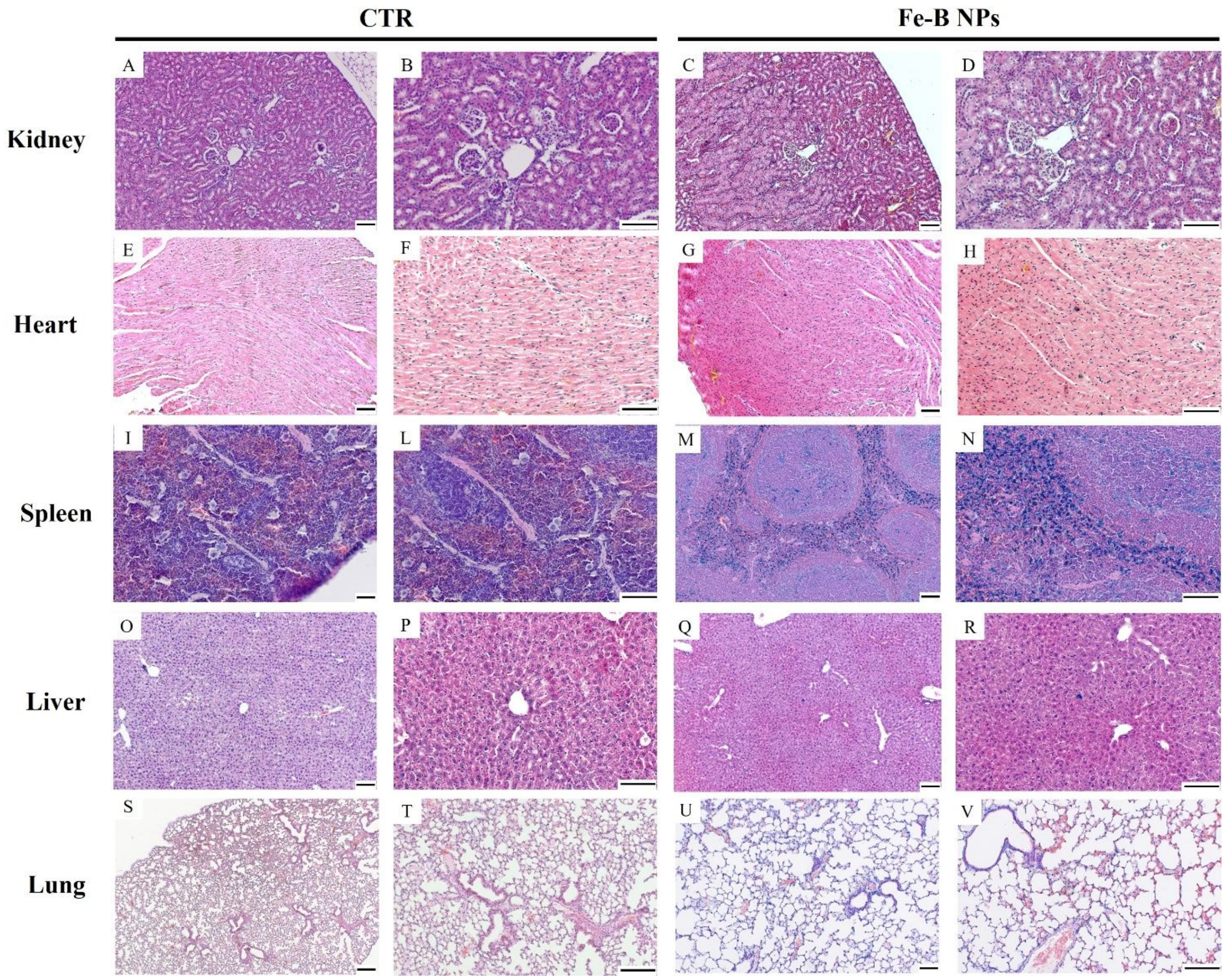

Figure S5. Histological evaluation of major organs following Fe-B NPs administration. Representative sections of kidney (A–D), heart (E–H), spleen (I–N), liver (O–R), and lung (S–V) from control (CTR) and NPs-treated mice stained with Prussian blue followed by hematoxylin and eosin (H&E). Blue deposits correspond to localized iron accumulation indicative of NPs presence (D, H, N, R), mainly detected in the liver and spleen. All organs display preserved morphology, with no evidence of necrosis, inflammation, or tissue degeneration, confirming that NPs are biocompatible and do not induce histopathological alterations. Images in columns 1 and 3 were acquired at 10× magnification, while those in columns 2 and 4 were acquired at 20× magnification. Scale bar = 100 µm.

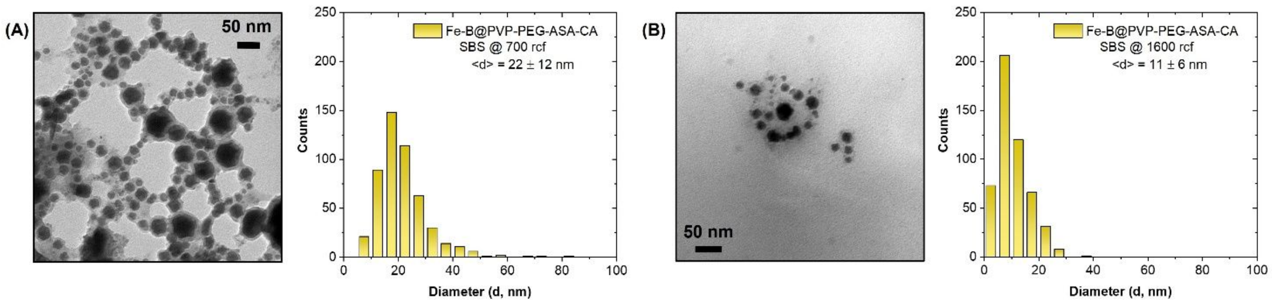

Figure S6. Representative TEM images and size distribution of Fe-B@PVP-PEG-ASA-CA NPs after a size refinement procedure based on SBS. The initial size distribution of 18 ± 18 nm is reduced to 22 ± 12 nm and 11 ± 6 nm for the samples collected at, respectively, 700 rcf (A) and 1600 rcf (B). Size statistics was performed on N > 500 NPs for each sample.

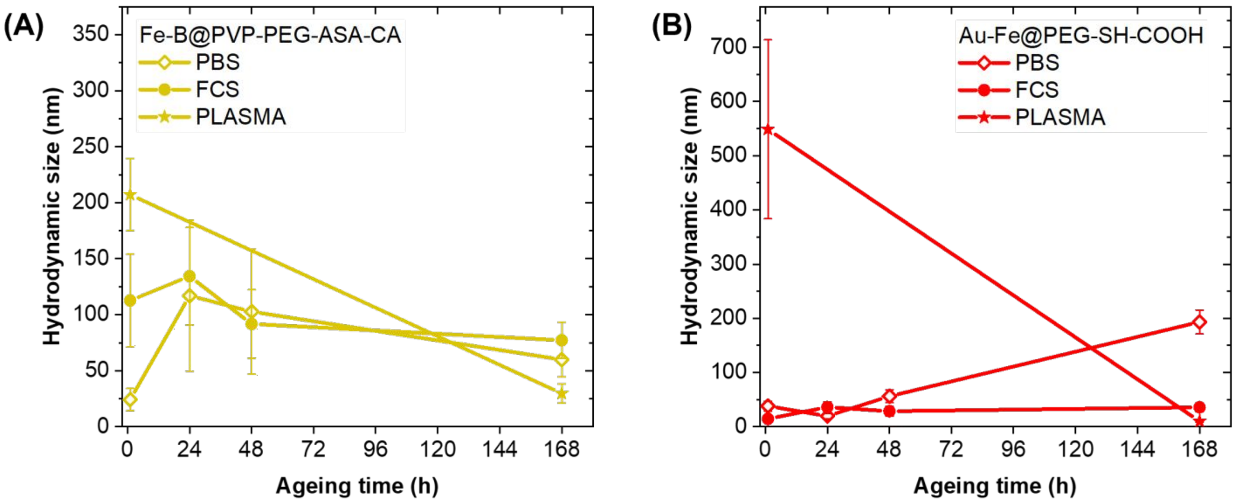

Figure S7. DLS analysis of Fe-B@PVP-PEG-ASA-CA (A) and Au-Fe@PEG-SH-COOH (B) after aging in PBS, FCS and plasma for 1 week.
